# Direct and Indirect Effects of Fe‐Incorporation in Nickel(oxy)hydroxide Materials for the Electrocatalytic Oxygen Evolution Reaction ‐ Employing Constant pH/U Models for Deeper Insights

**DOI:** 10.1002/chem.202501441

**Published:** 2025-09-09

**Authors:** Gustavo T. Feliciano, Kalishankar Bhattacharyya, Alexander A. Auer

**Affiliations:** ^1^ Department of Molecular Theory and Spectroscopy Max‐Planck‐Institut für Kohlenforschung 45470 Mülheim an der Ruhr Germany; ^2^ Chemistry Department Indian Institute of Technology Guwahati Assam 781039 India

**Keywords:** DFT, electrocatalysis, heterogeneous catalysis, oxygen evolution reaction, renewable energies, transition metal oxides

## Abstract

In this study, we seek to deepen the understanding of the Fe effect in Ni‐oxyhydroxide‐mediated oxygen evolution reaction (OER) electrocatalysis in alkaline conditions, where extremely small amounts of Fe can have a dramatic impact on catalytic performance. For this purpose, Density Functional Theory (DFT) electronic structure calculations with implicit solvation description is employed in a constant pH/potential simulation framework. Nanoparticle models are considered for the nickel‐based oxyhydroxide material with different degrees of Fe incorporation, and the pH/U‐dependent interface structure is studied. It can be seen that Fe incorporation influences the total extent of oxidation and deprotonation, stabilizing oxo species at early states of the reaction even at lower potentials.

From the resting state models, we derive reaction energy profiles and O‐O coupling barriers for three different OER mechanisms: water nucleophilic attack (WNA), intramolecular coupling (IMC), and the lattice oxygen mechanism (LOM). Each species is derived taking into account explicit change in protonation state and charge as a function of pH and potential. The results suggest direct and indirect modifications in Ni‐oxyhydroxide reactivity and in the preferred OER pathway, which changes with Ni/Fe ratio. The results we present imply that synergy between Ni and Fe acid‐base and redox properties is essential for efficient water oxidation/deprotonation and O‐O bond formation.

## Graphical abstract

In this work, the effect of iron incorporation in nickel oxide electrocatalysts for water oxidation is analyzed using electronic structure methods with implicit solvation and constant pH/potential methods. The location and amount of iron influences not only the involved deprotonation and oxidation processes but also the mechanism and nature of species in the O–O coupling reaction.

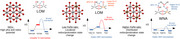

## 1 Introduction

Transition metal‐based oxide materials are promising catalysts for the oxygen evolution reaction (OER) in alkaline conditions, and some of them are widely studied, both experimentally and theoretically. Especially for nickel‐based materials, it has been reported that the presence of iron can dramatically change the OER activity of nickel‐containing stainless steel electrodes,^[^ ^1^, ^2^, ^3^, ^4^ ^]^ and in principle, even very small amounts of iron in the electrolyte solution can have sizable effects,^[^ ^5^, ^6^ ^]^ lowering the OER overpotential and increasing the current density. This is commonly referred to as the “Fe effect”. Many studies sought to clarify aspects of the Fe effect, especially in cobalt and nickel oxides. There are studies that suggest that Fe incorporation in NiOOH and specific Fe sites are responsible for OER catalysis, without effects from the Ni, in NiOOH.^[^ ^7^ ^]^ Liu et al. performed a detailed analysis of the dynamics of Fe adsorption/desorption in ${\mathrm{CoO}}_{x}$/${\mathrm{CoFeO}}_{x}$ thin films, suggesting that the sites with high OER activity are formed at the surface from Fe in solution.^[^ ^8^ ^]^ Ou et al. also studied the nature of the Fe site formation in Fe and Ni oxyhydroxides, arguing that the active Fe sites are formed at the surface, and at varying Fe concentrations, cooperative surface Fe–Fe adjacent sites are formed, which are determinant for OER performance.^[^ ^9^ ^]^ Trotochaud et al. observed changes in the activity of Ni oxyhydroxides, especially during Fe incorporation upon phase transformation of the material, and the OER activity was attributed also to charge transfer mediated activation effects of Fe over Ni.^[^ ^10^ ^]^ Further effects are unveiled when considering the influence of the pH. Fe induces early oxidation and deprotonation of the catalyst at high pH. The proton–electron transfer events become decoupled, and in principle more Ni sites can be oxidized and deprotonated.^[^ ^11^ ^]^ OER studies in NiOOH in Fe‐free electrolyte also highlight the importance of surface deprotonation for efficient OER, and suggest complex roles for the foreign metal.^[^ ^12^ ^]^ Even more recently, it has been pointed out that incorporation of Fe triggers electronic structure changes in the material, inducing deprotonation and oxidation in Fe‐bound oxygen atoms, but also affecting the reactivity of the atoms in its vicinity,^[^ ^13^ ^]^ and this effect can in principle depend on the relative Ni/Fe content.^[^ ^14^ ^]^

In order to get further insights about the role of Fe in NiOOH‐mediated OER catalysis, several computational studies have been carried out and published in recent years. Some studies suggest that Ni and Fe play synergistic roles during oxygen evolution reaction (OER).^[^ ^15^ ^]^ DFT and ab initio molecular dynamics (AIMD)‐based methods were employed for studying the effect of a nearby Fe atom in NiOOH, where proton coupled electron transfer (PCET) processes, involving the two metal atoms simultaneously can significantly stabilize reactive species for OER.^[^ ^16^ ^]^ Other mechanistic studies, including microkinetic modeling, suggest that different OER reaction mechanisms are coupled, with different contributions to the overall kinetic behavior, depending on potential and doping metal in NiOOH.^[^ ^17^ ^]^ Several other aspects of the reaction were unravelled by studies of our group in which we employed a description including solvation as well as pH and potential,^[^ ^18^ ^]^ where the Fe effect in ${\mathrm{CoO}}_{x}$ is analyzed. We found that the coexistence of species with different acid–base and redox properties is essential for the stabilization of critical OER intermediates.^[^ ^19^ ^]^ Furthermore, we complemented detailed experimental studies on thin Ni and NiFe oxide films with computational investigations that already indicated the special influence of Fe in these materials,^[^ ^13^, ^14^ ^]^ on which we will expand in the following.

In this study, we present a computational study of the effect of Fe incorporation into the surface structure of ${\mathrm{NiO}}_{x}$ and its electrocatalytic properties for the alkaline OER. This is done using electronic structure calculations together with a specific protocol for the inclusion of pH and potential effects. The extent of deprotonation/oxidation as well as the O‐O bond formation mechanism are analyzed and found to have a remarkable dependence on the relative Ni/Fe content, as well as the potential.

## 2 Results and Discussion

### 2.1 Catalyst Model Structures

**Figure 1:**
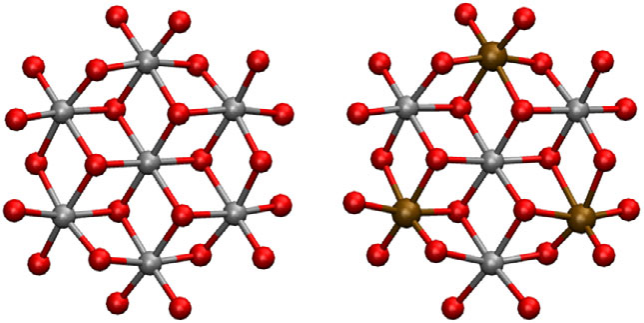
Illustration of the catalyst models employed in this study, showing the pure ${\mathrm{Ni}}_{7}O_{24}$ model and the modified ${\mathrm{Ni}}_{4}{\mathrm{Fe}}_{3}O_{24}$ model. Nickel atoms are depicted in silver, oxygen in red, and iron in dark orange.

In this section, the computational modeling results of the OER reaction on ${\mathrm{NiO}}_{x}$, and the corresponding iron‐modified material are presented. The model structure was chosen to be a metal oxide cluster extracted from the γ‐NiOOH phase structure, with the first coordination shell of Ni atoms around the central Ni atom, yielding a ${\mathrm{Ni}}_{7}O_{24}$ nanoparticle (see Figure 1). The aqueous solvent is described by an implicit solvation model. This choice allows us to take into account the effect of several metal centers and their chemical environment on reactivity while retaining computational efficiency. Note that in order to assess convergence of the results with system size, OER profiles have also been computed for a ${\mathrm{Ni}}_{19}O_{54}$ model, for which the same rate‐limiting step assignment and a similar charge/protonation distribution profile has been obtained (see Supporting Information for details).

**Figure 2:**
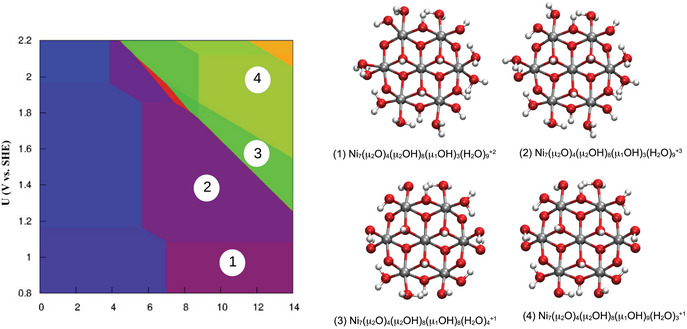
Computational Pourbaix diagram for initial screening of the relevant structures accessible in alkaline conditions.

For the computational modeling of the OER reaction on the relevant metal oxides, the pH and potential are important parameters that dictate the system protonation and total charge state. In order to take these effects into account, each model structure is constructed by starting from a structure with the highest amount of protons and highest degree of reduction. Next, new structures are generated and optimized by successive removal of protons and electrons. The energy contribution of the removed protons and electrons is evaluated using an implicit energy reference for electrons (SHE) and the proton solvation energy and chemical potential at a given pH, as performed in previous studies.^[^ ^18^, ^19^, ^20^ ^]^ At each pH/U value, the structure with the lowest free energy is selected, resulting in a 2D‐pH/U “computational Pourbaix diagram”. In Figure 2, the pH/U diagram for ${\mathrm{Ni}}_{7}O_{24}$ is shown, and 4 different areas can be identified close to pH 14. Using this information, the final model structure used in further simulations is selected, ensuring that it (within the computational model) resembles a state in equilibrium with an electrode at a given electrochemical potential and a solvent with a given pH.

**Figure 3:**
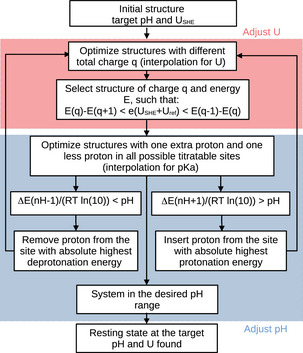
Flowchart illustrating the refinement procedure, corresponding to the pH/potential equilibration protocol.

A subsequent refinement protocol is then applied, in which more oxidation/protonation state changes are performed, until all the remaining protons in the system have pKa values higher than 14. This way, the resulting structure represents a system in equilibrium with an aqueous solution at pH 14, as well as an electrode at a given potential. We will refer to this “equilibration” of electrons and protons as the pH/potential protocol in the following, and the framework is better illustrated in Figure 3.

**Figure 4:**
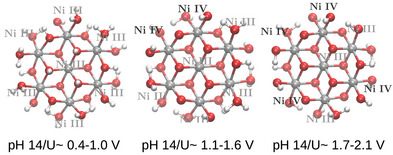
Structures from the ${\mathrm{Ni}}_{7}O_{24}$ model obtained from the pH/potential refinement protocol, applied to the structures 2, 3, and 4 from the computational Pourbaix diagram, illustrated in Figure 2, together with the respective potential range and pH value where they are obtained.

This protocol is applied to the structures corresponding to the regions 2, 3 and 4 of the pH/U diagram, and the resulting structures are shown in Figure 4. For the sake of comparing the degree of oxidation of the metal centers, an oxidation state assignment is carried out, based on the metal‐oxygen coordination geometry, specifically regarding metal‐oxygen bond lengths. As it can be seen, an increasing number of metal centers undergo ${\mathrm{Ni}}^{III}$ to ${\mathrm{Ni}}^{IV}$ oxidation with increasing potential. At the lowest potential range, U = 0.4–1.1 V, the degree of protonation of the oxygen atoms is very high: most of the $\mu_{1}$ ligands are ${\mathrm{OH}}_{2}$ (only three of them are in OH form), and all $\mu_{2}$/$\mu_{3}$ oxygens are protonated. Going to 1.1–1.6V, deprotonation of all $\mu_{3}$ sites and a few $\mu_{2}$ sites is observed, and the $\mu_{1}$ oxygen atoms from adjacent Ni center pairs, on the edge of the cluster (referred to as a “Ni–Ni” reaction site), are in the *${\mathrm{OH}}_{2}$*OH configuration (referring to the parallel $\mu_{1}$ oxygens in the Ni–Ni site). Furthermore, more $\mu_{1}$ oxygen atoms are deprotonated in the 1.7‐2.1V range, and the first *OH*OH pairs are formed, as further Ni centers are oxidized. These findings correlate well with experimental EXAFS measurements, and the deduced structural changes and oxidation state evolution with increasing potential.^[^ ^21^, ^22^ ^]^

**Figure 5:**
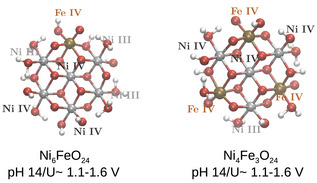
Structures from the ${\mathrm{Ni}}_{6}{\mathrm{FeO}}_{24}$ and ${\mathrm{Ni}}_{4}{\mathrm{Fe}}_{3}O_{24}$ model obtained from the pH/potential refinement protocol, together with the respective potential range and pH value where they are obtained.

In order to access the effect of iron incorporation, the ${\mathrm{Ni}}_{7}O_{24}$ structures were used as a starting point, and two possibilities were considered, regarding the number of Fe atoms: (i) ${\mathrm{Ni}}_{6}{\mathrm{FeO}}_{24}$, which allows us to identify direct and indirect effects of iron with the lowest Fe amount (ii) ${\mathrm{Ni}}_{4}{\mathrm{Fe}}_{3}O_{24}$, which corresponds to the Fe/Ni ratio closest to the experimentally reported ratio with the best OER performance. ${\mathrm{Ni}}_{6}{\mathrm{FeO}}_{24}$ and ${\mathrm{Ni}}_{4}{\mathrm{Fe}}_{3}O_{24}$ models are obtained from the pH/potential‐optimized ${\mathrm{Ni}}_{7}O_{24}$ structures by substitution of Ni atoms with Fe. The location of the substitution is chosen by evaluating the energy of all possible Fe arrangements in the cluster structure. We observed that Fe replacement is always energetically favorable at the edge sites of the cluster, and in the ${\mathrm{Ni}}_{4}{\mathrm{Fe}}_{3}O_{24}$ structure, with the Fe atoms as far away from each other as possible (note that a detailed analysis is reported in reference 14). The resulting model structures are shown in Figure 5. It can be noted that all Fe centers are already oxidized, and exhibit notably short Fe─O bond lengths, for both Fe/Ni ratios considered. Particularly in the ${\mathrm{Ni}}_{4}{\mathrm{Fe}}_{3}O_{24}$, all Fe centers contain an oxo group, and there are more ${\mathrm{OH}}_{2}$ ligands bound to the edge Ni centers, compared to ${\mathrm{Ni}}_{7}O_{24}$ in the same potential range.

### 2.2 OER Reaction Path and Energy Profile

From the initial state of each model, the OER reaction energy path is computed. In our model, the reactive site is always regarded as an adjacent pair of metal atoms at the edge of the nanoparticle, and therefore, there are six possible reaction sites. We focus on one reaction site, and it is chosen as the site with lowest energy change upon proton removal (and potentially an electron removal). The pH/U protocol is again applied to the rest of the structure, to ensure that it still corresponds to the equilibrium condition with the electrode potential and pH 14. The initial configuration of the reactive center, at pH 14 and U = 1.1–1.6V is considered to be, in all cases, *${\mathrm{OH}}_{2}$*OH. After the first $H^{+}$/$e^{-}$ removal, the possibilities are *OH*OH or a *${\mathrm{OH}}_{2}$*O, and finally, another $H^{+}$/$e^{-}$ pair removal generates the *OH*O configuration.

**Figure 6:**
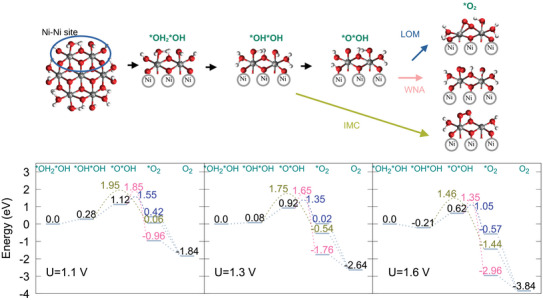
Structural models for OER reaction intermediates for ${\mathrm{Ni}}_{7}O_{24}$, highlighting the Ni–Ni active site (upper panel). Corresponding OER energy profile for different potentials. Results for the reaction energy barriers for each mechanism and *$O_{2}$ intermediate energies are depicted in green for IMC, pink for WNA and blue for LOM (lower panel).

**Figure 7:**
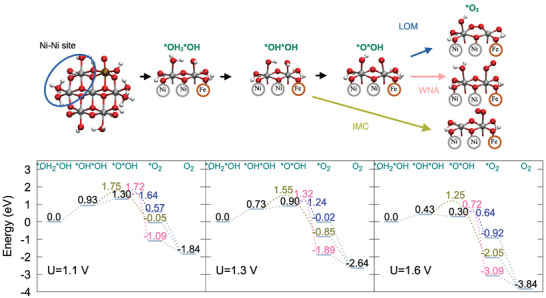
Structural models for OER reaction intermediates for ${\mathrm{Ni}}_{6}{\mathrm{FeO}}_{24}$, highlighting the Ni–Ni active site (upper panel). Corresponding OER energy profile for different potentials. Results for the reaction energy barriers for each mechanism and *$O_{2}$ intermediate energies are depicted in green for IMC, pink for WNA and blue for LOM (lower panel).

**Figure 8:**
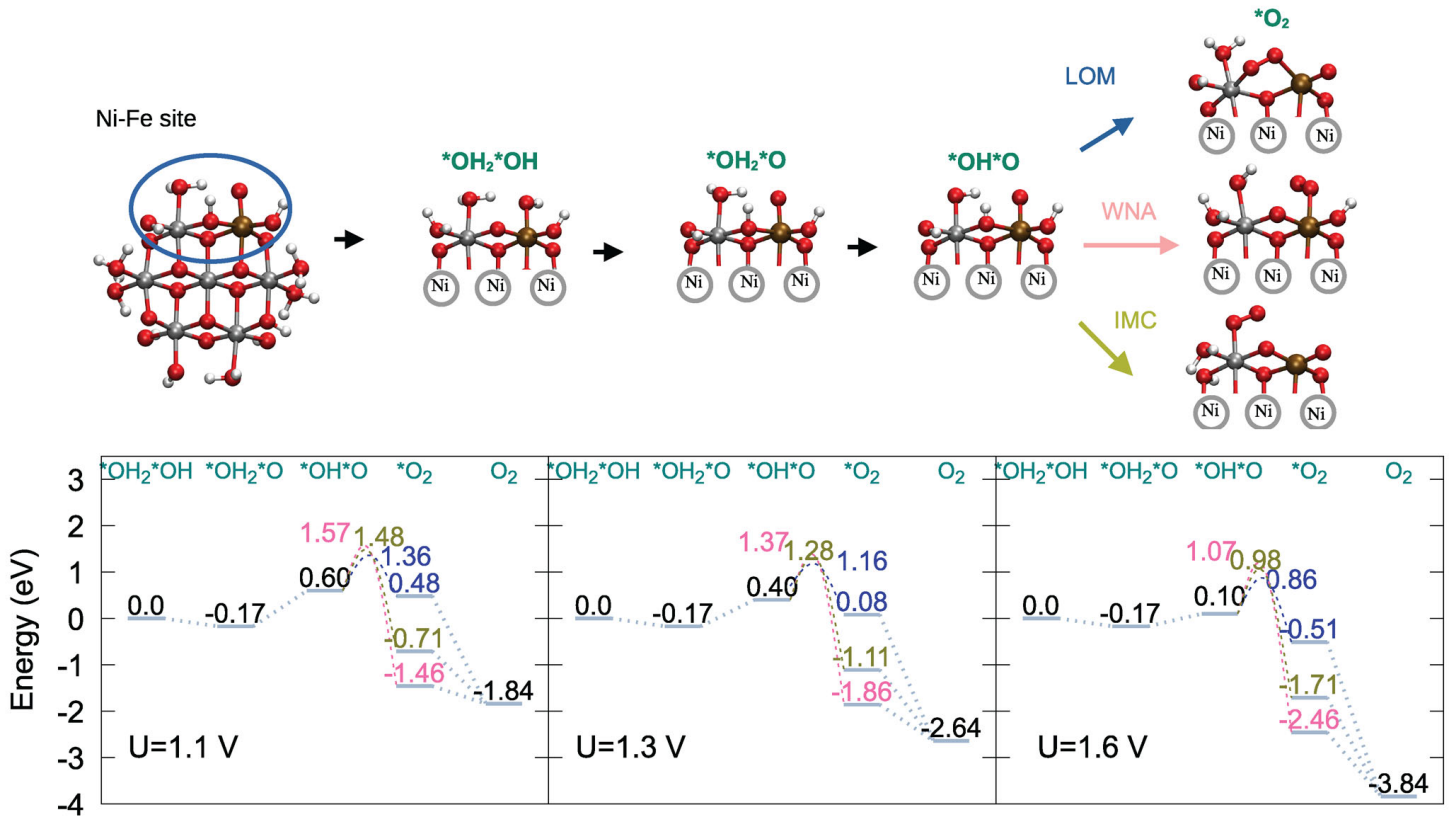
Structural models for OER reaction intermediates for ${\mathrm{Ni}}_{6}{\mathrm{FeO}}_{24}$, highlighting the Ni–Fe active site (upper panel). Corresponding OER energy profile for different potentials. Results for the reaction energy barriers for each mechanism and *$O_{2}$ intermediate energies are depicted in green for IMC, pink for WNA and blue for LOM (lower panel).

**Figure 9:**
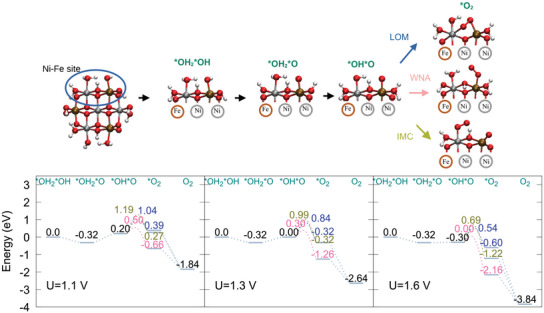
Structural models for OER reaction intermediates for ${\mathrm{Ni}}_{4}{\mathrm{Fe}}_{3}O_{24}$, highlighting the Ni–Fe active site (upper panel). Corresponding OER energy profile for different potentials. Results for the reaction energy barriers for each mechanism and *$O_{2}$ intermediate energies are depicted in green for IMC, pink for WNA and blue for LOM (lower panel).

In the O–O coupling reaction, three mechanisms are possible: i) lattice oxygen mechanism (LOM), when coupling occurs between a $\mu_{1}$ and a $\mu_{2}$ oxygen, ii) intramolecular coupling (IMC), between $\mu_{1}$ oxygens, and iii) water nucleophylic attack (WNA), involving a catalyst oxygen atom and a water molecule oxygen atom. The OER reaction intermediate structures, together with the OER energy path at different potentials, and the lowest activation energies for the O─O bond formation are shown in Figures 6, 7, 8, 9. The O–O coupling transition state (TS) energies for the different mechanisms are reported with respect to the initial intermediate free energy. Note that there are no changes in the total charge and protonation state in the energy profile as obtained from a Nudged Elastic Band (NEB) transition state search. Only the initial structure and the product from the NEB are subjected to the pH/U refinement procedure, yielding the structure and energy of the *$O_{2}$ intermediate, which is different for each studied mechanism. The energy of the OER endpoint is reported as the energy of the semi‐reaction 2$H_{2}O$ ⟶ $O_{2}$ + 4$H^{+}$ + 4$e^{-}$.

${\mathrm{Ni}}_{7}O_{24}$ **– Ni‐Ni site** Before the OER onset, at U = 1.1 V, (Figure 6, left panel), the *OH*OH intermediate has a free energy of 0.28 eV, and the *O*OH intermediate has a free energy of 1.12 eV, indicating that even the initial deprotonation events are unfavorable. The transition state for the O‐O coupling step in the IMC mechanism (green, Figure 6) lies at 1.95 eV, followed by 1.85 eV for WNA (pink, Figure 6) and 1.55 eV for LOM (blue, 6). Furthermore, the *$O_{2}$ intermediate shows even a positive free energy in some of the mechanisms (0.42 eV for LOM and 0.06 eV for IMC).

When increasing the potential to 1.3 V (Figure 6, central panel), the *OH*OH intermediate becomes more accessible, but *O*OH still has a free energy of 0.92 eV. The IMC barrier is the highest barrier (1.75 eV), yielding the *$O_{2}$ intermediate in the superoxide form, where $O_{2}$ is bound to one metal center and the adjacent metal center is now pentacoordinated. The IMC *$O_{2}$ intermediate free energy lies at ‐0.54 eV, despite the energy penalty of the metal center coordination changes. Although the transition state energies of O–O coupling for WNA and LOM are still high (1.65 and 1.35 eV, respectively), most of the associated free energy change is attributed to the formation of the *O*OH intermediate. The LOM *$O_{2}$ intermediate has a free energy of 0.02 eV, which is also a consequence of the reactive center distortion, affecting the coordination sphere of both Ni centers. The WNA *$O_{2}$ reaction intermediate always has the lowest free energy (‐1.76 eV), compared to the same intermediate in the other mechanisms. This is possibly due to two reasons: i) the proton transfer from the incoming water molecule to the OH group in the reaction center is exergonic, and this intermediate is found in the superoxide form. ii) As a consequence, formation of the *$O_{2}$ intermediate occurs with much less distortion of the coordination sphere of the metal atoms at the reaction center.

At U = 1.6 V (Figure 6, right panel), the *OH*OH intermediate shows a negative free energy (‐0.21 eV), while *O*OH intermediate still lies at 0.62 eV, and it is the rate‐limiting step. In the IMC mechanism, the reaction barrier for O‐O coupling is still the highest (1.46 eV), and the LOM still displays the TS with the lowest energy (1.05 eV). The TS energy reduction for LOM and WNA mechanisms stems from the fact that the *O*OH intermediate is obtained with the lowest free energy possible at this potential.

${\mathrm{Ni}}_{6}{\mathrm{FeO}}_{24}$ **– Ni–Ni site** The Fe effect is experimentally reported to occur with very small amounts of Fe in the electrolyte. Therefore, it is possible that iron indirectly changes the reactivity at a Ni center, but it is also possible that the OER occurs directly at a Fe center. For the chosen Fe/Ni ratio in the simulation model, we analyze both possibilities, and separately discuss the effects of iron incorporation in the ${\mathrm{NiO}}_{x}$ structure and the OER energy profile for both cases. In this paragraph, we describe the “indirect” Fe effect, and thus, the reaction intermediates are considered to be formed at two adjacent Ni atoms, in direct vicinity of a Fe atom (see Figure 7).

At U = 1.3 V (Figure 7, center panel), it can be seen that the initial deprotonation of the *${\mathrm{OH}}_{2}$*OH is even more difficult, when compared to the ${\mathrm{Ni}}_{7}O_{24}$ case. As discussed in the previous sections, the oxygen atoms at the Fe center exhibit a higher deprotonation extent, which affects the acidity of oxygen atoms at other Ni atoms. The formation of the *O*OH intermediate is also endergonic (0.90 eV), close to the free energy change in ${\mathrm{Ni}}_{7}O_{24}$ (0.92 eV at U = 1.3 V). The reaction barriers for all mechanisms, as well as the *$O_{2}$ intermediate energies follow the same trend observed in ${\mathrm{Ni}}_{7}O_{24}$, although the reaction barriers are notably smaller (0.1–0.3 eV), which is already a strong indication of a significant indirect Fe effect.

At U = 1.6 V (Figure 7, right panel), the OER profile shows that the *O*OH is strongly stabilized (0.30 eV vs. 0.62 eV in ${\mathrm{Ni}}_{7}O_{24}$ at 1.6V), more than the *OH*OH intermediate (0.43 eV), and now the first deprotonation is the rate‐limiting step. Ni centers close to Fe display different acidity properties, as the Fe center is oxidized. Therefore, the H+/e‐ transfer events in the first reaction steps take place with a different proton redistribution pattern, compared to ${\mathrm{Ni}}_{7}O_{24}$. These factors ultimately affect the *O*OH formation free energy. The LOM and WNA show a sensible drop in the TS energy (0.64 eV for LOM and 0.72 for WNA) compared to ${\mathrm{Ni}}_{7}O_{24}$, due to stabilization of the *O*OH intermediate, and also for the O–O coupling energy barrier. The *$O_{2}$ intermediate has lower free energies for all mechanisms, compared to ${\mathrm{Ni}}_{7}O_{24}$, especially in the IMC mechanism.

${\mathrm{Ni}}_{6}{\mathrm{FeO}}_{24}$ **– Ni–Fe site** In this paragraph, the OER reaction intermediates are analyzed when the reaction takes place at a Ni–Fe reaction center. The energy profile and structures (Figure 8) show that the deprotonation of the *${\mathrm{OH}}_{2}$*OH generates the *${\mathrm{OH}}_{2}$*O intermediate, instead of *OH*OH, meaning that the oxygen atom at the Fe center is first fully deprotonated before the ${\mathrm{OH}}_{2}$ ligand at the neighboring Ni center. The *${\mathrm{OH}}_{2}$*O intermediate formation is exergonic by 0.17 eV for all potentials, and it corresponds to the OER resting state. The deprotonation of oxygen at the Ni atom is now the rate‐limiting step, generating the *OH*O intermediate. When analyzing the profile at U = 1.3 V (Figure 8, center panel), it can be seen that the deprotonation of the oxygen at the Ni atom in the Ni–Fe center is still endergonic (0.40 eV). The transition states for O–O coupling now show different trends: WNA displays the TS with the highest energy (1.37 eV), while IMC TS shows a significant energy reduction, to 1.28 eV, and LOM TS has a slight energy reduction, to 1.16 eV.

In the OER energy profile at U = 1.6 V (Figure 8, right panel), further stabilization of the *OH*O intermediate is observed, and the respective energy is now 0.10 eV. The LOM pathway is still the preferred one, regarding the overall energy change (TS of 0.86 eV), but the associated *O2 intermediate is still the highest in energy, due to the already observed distortion at the reactive center. By comparing the Ni–Fe and Ni–Ni reaction center profiles at U = 1.6 V in ${\mathrm{Ni}}_{6}{\mathrm{FeO}}_{24}$, it can be noted that the formation of the initial intermediates by deprotonation/oxidation are thermodynamically much more accessible at the Ni–Fe center (formation energy of the first two intermediates is 0.43/0.30 eV for Ni–Ni and ‐0.17/0.10 eV for Ni–Fe), while the energy barriers for O–O coupling are significantly lower at the Ni–Ni site (LOM/WNA barriers are 0.34/0.42 eV for Ni‐Ni versus 0.76/0.97 eV for Ni‐Fe).

${\mathrm{Ni}}_{4}{\mathrm{Fe}}_{3}O_{24}$ **– Ni–Fe site** In this paragraph, the OER reaction intermediates are analyzed when the reaction takes place at a Ni–Fe reaction center. At U = 1.3 V (Figure 9, center panel), the free energy of all intermediates prior to the O–O coupling step is essentially the same. This corresponds to the first situation where, at the intermediate potential, the entire OER profile has intermediates with equal or lower free energy than the initial state. This already indicates a much lower overpotential than in the ${\mathrm{Ni}}_{7}O_{24}$ materials with low Fe content. The oxo intermediate is therefore more strongly stabilized, compared to the ${\mathrm{Ni}}_{6}{\mathrm{FeO}}_{24}$ case. Deprotonation of the Ni center, in vicinity of the Fe center is also more strongly favored.

At U = 1.6 V (Figure 9, right panel), the *OH*O intermediate is even further stabilized, with a free energy of ‐0.30 eV. Due to the short Fe‐O bond length observed in the oxo group, the energetic cost for O–O coupling involving the Fe bound oxygen atom and an adjacent $\mu_{1}$ or $\mu_{2}$ oxygen atom is still relatively high, as shown in the IMC and LOM energy barriers, just like the OER profile at the Ni–Fe center, for the ${\mathrm{Ni}}_{6}{\mathrm{FeO}}_{24}$ model. Nevertheless, the WNA has the TS with the lowest energy (0.30 eV), and it corresponds to the lowest energy barrier among all models and mechanisms studied. Our results suggest that there is a synergy between direct and indirect effects of Fe. A reactive oxo species is generated at the Fe center as a direct effect. At the same time, there is a change of the acidity/redox properties of the nearby Ni atom. This results in a favorable scenario for the nucleophilic attack of a water molecule on the oxo group.

In conclusion, for ${\mathrm{Ni}}_{7}O_{24}$ at pH 14/U = 1.6V, at the Ni centers, formation of the *OH*OH/*O*OH species has a free energy of ‐0.21/+0.62 eV. The *OH*OH intermediate can be accessed by deprotonation of a $H_{2}O$ group. The quick hydrogen bond network reorganization, together with oxidation of a Ni center can stabilize this intermediate. The *O*OH intermediate, however, is not easily stabilized, as the pKa of the M‐OH group is significantly higher. Oxidation of another Ni center usually cannot compensate for the required energy, unless the electrode potential is further increased. The results also show that the reaction barrier for LOM is the lowest among the studies mechanisms.

The iron replacement in the ${\mathrm{NiO}}_{x}$ structure causes several important changes. Indirectly, the presence of Fe can change the reactivity of Ni centers close to it. Interestingly, the *OH*OH intermediate is destabilized, while *O*OH is stabilized compared to ${\mathrm{NiO}}_{x}$, with free energies of +0.43/+0.30 eV. The first deprotonation event is actually disfavored in the presence of Fe. The deprotonation extent is always locally higher in the Fe coordination environment, where oxidation also happens first, hindering further deprotonation of the oxygen groups at the Ni centers. On the other hand, in the next deprotonation, the Ni center adjacent to the Fe center becomes more readily oxidizable, and a coupled deprotonation/oxidation step stabilizes the *O*OH intermediate. This also generates a very reactive Ni–O species, which exhibits lower reaction energy barriers for the WNA/LOM mechanisms.

As a direct effect, *${\mathrm{OH}}_{2}$*O/*OH*O intermediates are strongly stabilized at the Fe center, with a free energy of ‐0.17/+0.10 eV, leading to iron‐oxo species at early stages of the reaction. The hydrogen bonding network between the Fe–O group and the Ni‐${\mathrm{OH}}_{2}$ group is particularly strong. As the pKa of the Fe‐oxo group is slightly below 14, it can further help in the release of one proton from the Ni‐aquo group, which helps to stabilize the *OH*O intermediate. However, reaction barriers for O─O bond formation are significantly higher, compared to the reaction at the Ni–Ni site. As the Fe center is oxidized, and the deprotonation extent is large, we observe higher reaction barriers at the Fe═O group for the WNA/LOM mechanisms.

When increasing the Fe content, at pH 14/U = 1.6 V, the *${\mathrm{OH}}_{2}$*O/*OH*O intermediates are downhill in energy (free energy of ‐0.32/‐0.30eV), and the reaction barrier is the lowest at the same reaction site for the WNA mechanism. The oxo group is even more stabilized, compared to all previous cases, and the adjacent Ni center is more easily oxidized, and its respective oxygen ligands deprotonated. The pKa of the OH group at the Ni center facilitates the deprotonation of the incoming water molecule in the presence of a reactive Fe–O species.

### 2.3 Detailed Analysis of the Fe Effect

In the previous sections several characteristic properties were found: Fe does enhance OER significantly and increasingly so, if the Fe content is increased. From the studied models two basic effects can be observed ‐ Fe is oxidized at much lower potentials than Ni and oxidized Fe centers have an effect on adjacent Ni atoms. Furthermore, oxygen species bound to Fe are much more acidic and will be deprotonated more easily compared to Ni, which has an effect on the hydrogen bond network in the vicinity of a Fe center. In this section, additional analyses are performed to dissect specific effects of the Fe incorporation to the OER efficiency in NiOOH.

As a first step, the oxidation and deprotonation free energies are analyzed separately for the *${\mathrm{OH}}_{2}$*OH and *OH*OH intermediates in ${\mathrm{Ni}}_{7}O_{24}$ and the *${\mathrm{OH}}_{2}$*OH and *${\mathrm{OH}}_{2}$*O intermediates in ${\mathrm{Ni}}_{4}{\mathrm{Fe}}_{3}O_{24}$ at pH 14 and U = 1.6 V. This sheds some light on whether the Fe effect is mostly a change in acidity of the involved hydroxy species like it was reported for CoOx systems,^[^ ^19^ ^]^ or whether the redox properties of the material are significantly altered by the presence of Fe.

**Figure 10:**
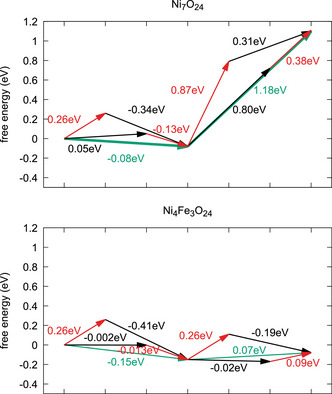
PCET diagrams for ${\mathrm{Ni}}_{7}O_{24}$ and ${\mathrm{Ni}}_{4}{\mathrm{Fe}}_{3}O_{24}$, depicting PCET (green arrows), deprotonation (red arrows) and oxidation (black arrows) processes. Y‐axis denotes the free energy, free energy differences are denoted on the arrows.

The free energy changes are illustrated as a vector diagram in Figure 10, where green arrows denote the net PCET processes, red arrows denote proton transfer (deprotonation), and black arrows denote electron transfer (oxidation). A comparison of the vector diagrams without (left panel) and with (right panel), Fe clearly shows how large the impact of Fe is: for both reaction steps the free energy required for the redox as well as the deprotonation step are significantly lowered. The same trend can be observed for ${\mathrm{Ni}}_{6}{\mathrm{FeO}}_{24}$ (PCET diagram supplied in the Supporting Information). Hence, we will now look at both effects in detail separately, starting with the changes in oxidation behavior upon Fe inclusion.

**Figure 11:**
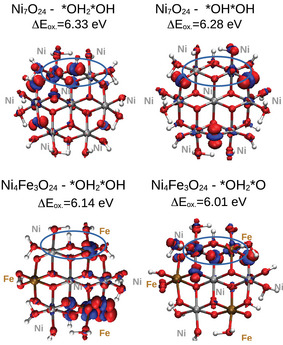
Electron density difference isosurfaces upon vertical one‐electron removal for the first and second OER intermediates in ${\mathrm{Ni}}_{7}O_{24}$ and ${\mathrm{Ni}}_{4}{\mathrm{Fe}}_{3}O_{24}$, along with the corresponding energies. Regions of electron accumulation are depicted in blue (isovalue 0.004 e.${\mathrm{Bohr}}^{-3}$) and electron depletion in red (isovalue ‐0.004 e.${\mathrm{Bohr}}^{-3}$).

Figure 11 displays the electron density difference isosurfaces for the OER intermediates in ${\mathrm{Ni}}_{7}O_{24}$ and ${\mathrm{Ni}}_{4}{\mathrm{Fe}}_{3}O_{24}$. In this plot, we do a subtraction between the charge density volumetric map of an OER intermediate and its oxidized form, employing the same geometry. From this information, the influence of Fe on the electronic reorganization upon oxidation can be analyzed. The energy difference between the corresponding OER intermediate and its oxidized form is also reported.

The results show that the oxidation energy is always lower in the presence of Fe. Moreover, in the ${\mathrm{Ni}}_{7}O_{24}$ case, the first oxidation event involves reorganization of Ni 3d orbitals, but mainly O 2p orbitals in the second. For ${\mathrm{Ni}}_{4}{\mathrm{Fe}}_{3}O_{24}$, the first oxidation causes changes mostly at Fe centers, but the second oxidation involves Ni‐3d and Fe‐3d adjacent centers. Hence, the results suggest that the presence of Fe can also induce oxidation of Ni centers, and as a consequence, non‐local changes in acidity can be expected. Furthermore, changes in the electron density upon oxidation in ${\mathrm{Ni}}_{4}{\mathrm{Fe}}_{3}O_{24}$ suggest that formation of open‐shell species takes place at neighboring metal centers and therefore, can facilitate the formation of triplet dioxygen. Note that a similar influence of the electronic spin density on reactivity was previously reported on single‐, dual‐ and few‐atom cluster actives sites for the ORR,^[^ ^23^ ^]^ and a similar influence has been reported for the ${\mathrm{CoO}}_{x}$ catalyzed OER, where the oxyl radical character correlates with the O–O coupling reaction barrier when Fe is present.^[^ ^19^ ^]^

**Figure 12:**
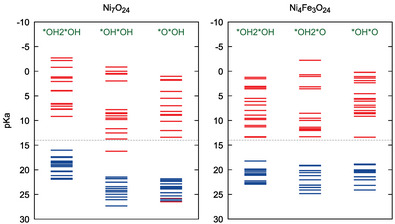
proton‐DOS of ${\mathrm{Ni}}_{7}O_{24}$ model (left) and ${\mathrm{Ni}}_{4}{\mathrm{Fe}}_{3}O_{24}$ (right). The structures are taken from the OER onset potential region.

In order to assess the effect of Fe on the acidity of the protons close to the active site, Figure 12 displays what we term as “protonation density of states” (pH‐DOS). Note that Cheng et al.^[^ ^24^ ^]^ discuss a similar approach in the framework of computing PCET steps for water oxidation. In this scheme, the pKa of each titratable site in the oxide is evaluated and put in the diagram as a horizontal line. The blue lines denote pKa values of the occupied sites (protonated) and red lines denote unoccupied sites (deprotonated), very much like the common density of states for occupied and unoccupied electronic levels / orbital energies. In this sense, the pH 14 value resembles an analogous “Brønsted‐level” for protons, in correspondence to the Fermi‐level for electrons (dashed gray line in Figure 12). The pH‐DOS diagram is evaluated for each reaction intermediate prior to the O–O coupling reaction, which nicely depicts the evolution of the acidity and redox potential as the reaction progresses, and how or many electrons and protons are energetically available for a given pH. This hints to the reason of the additional hurdles in further deprotonation/oxidation of the material and how the degree of Fe incorporation aids to overcome it.

In absence of Fe (Figure 12, left panel), the *OH*OH and *O*OH intermediates exhibit unoccupied sites with pKa values above 14 (note the inverse scale on graph), seen as red lines in the lower part. These sites correspond to sites in the active region, namely: i) one *OH site in *OH*OH, coming from the deprotonation of the *${\mathrm{OH}}_{2}$*OH and ii) the two sites in *OH*O. This means that the pH/U conditions are not sufficient to stabilize these intermediates, with a resulting increasing deprotonation energy penalty. This observation is in line with what is observed in the OER profile (see Figure 6), where the *OH*OH intermediate is only slightly stabilized by increasing potential, and the *O*OH intermediate has always positive formation free energy.

In the presence of Fe (Figure 12, right panel), we see that all intermediates are stabilized upon deprotonation. Moreover, the unnocupied site with pKa slightly below 14 corresponds to the μ1 oxygen site from Ni directly adjacent to the Fe‐oxo species. This configuration would be optimal for the WNA reaction mechanism, where water can be deprotonated by a basic species, while attacking the Fe‐oxo site. All these features can be also directly related to the OER profile (See Figure 9).

The combined analysis results strongly suggest that the increased ability to oxidize the material upon insertion of Fe explains the increase in the OER performance in NiOOH. The relative amount of Fe also controls how many electrons can be extracted, as well as the resulting acidity of the oxygen atoms. This leads to a significantly enhanced tendency of the system to undergo the essential oxidation and deprotonation steps when forming the O─O bond during the OER. This has also been found in a combined experimental and computational study on the changes in electronic structure that occur upon Fe incorporation, in which the results of x‐ray absorption, electrochemical testing and DFT calculations before and after activation of NiFe oxide catalyst layers are discussed.^[^ ^13^, ^14^ ^]^

A comparison with a previous study on the Fe effect in ${\mathrm{CoO}}_{x}$/${\mathrm{CoFeO}}_{x}$ materials^[^ ^19^ ^]^ shows that the OER intermediates are significantly more stable at lower pH and potential conditions for ${\mathrm{CoO}}_{x}$ than ${\mathrm{NiO}}_{x}$. As Co has a lower oxidation potential than Ni, replacement of one Co atom with Fe also enhances the reactivity and further stabilizes *OH*OH/*O*OH intermediates by increasing the acidity of the system. In the Ni/Fe system, we observe the same enhancement, due to oxidizability increase.

## 3 Conclusion

In this study, simulations for the OER on nickel oxide and nickel–iron oxide were performed using a series of cluster models. A computational scheme has been applied that allows to simulate conditions for pre‐ and post OER onset potentials as well as the protonation state of the interface in alkaline conditions. The OER reaction pathway is described for all deprotonation/oxidation intermediates and the rate‐limiting steps. Reaction barriers for the O–O coupling reaction are also computed for each of the three possible reaction mechanisms.

Our results clearly show that incorporation of Fe significantly stabilizes the OER intermediates prior to the O–O coupling reaction at Fe and Ni sites. Moreover, an increasing amount of Fe enhances the degree of oxidation and deprotonation of the interface, influencing the mechanism of the O─O bond formation. At low Fe concentrations, OER intermediates at a Fe site are more stable then at a Ni site, but also less reactive toward the O–O coupling reaction, which occurs preferentially by the lattice oxygen mechanism. At 1:1 Ni/Fe ratio, we have both high stabilization of OER intermediates and the lowest O─O bond formation barrier, for the water nucleophilic attack mechanism.

These results imply that, in NiOOH, Fe induces early oxidation, which increases the acidity of the oxygen groups as a consequence, and stabilizes the oxyl group. This is in contrast to what is observed in CoFeOOH materials, where the presence of Fe mostly increases acidity, leading to oxidation of the metal centers. As Fe can induce both, electron and proton removal, it can leverage the process that is most hindered in OER on a specific transition metal oxide.

The general approach applied here can be used for a detailed atomistic understanding for highly relevant systems with different metal compositions, which helps to rationalize and improve how multimetallic OER catalysts can be used to reduce energy losses in water electrolysis.

## 4 Computational Methods

The electronic structure calculations were performed using the ORCA 6.0 package.^[^ ^25^, ^26^ ^]^ The spin unrestricted generalized gradient approximation Perdew–Burke–Ernzerhof was employed,^[^ ^27^ ^]^ together with the def2‐SVP basis set^[^ ^28^, ^29^ ^]^ and the D3 dispersion correction with Becke–Johnson damping.^[^ ^30^ ^]^ In order to assess the consistency of the results with respect to the functional used, selected reactions and models were also computed using the B3LYP functional,^[^ ^31^, ^32^ ^]^ confirming that the qualitative conclusions are not affected by functional deficiencies like self‐interaction errors (see Supporting Information). We also employ the polarizable continuum model C‐PCM^[^ ^33^, ^34^ ^]^ for describing the aqueous environment. Although this is a very simple choice for the representation of the aqueous environment, it allows for the sampling of several protonation and charge configurations to capture the main effects of potential. Note that previous studies demonstrate the efficiency and validity of this approximation in comparison to other approaches.^[^ ^13^, ^14^, ^18^, ^19^, ^35^ ^]^ SCF convergence is improved though the use of the Trust Augmented Radius Hessian (TRAH) pseudo second‐order optimizer, implemented in ORCA.^[^ ^36^ ^]^ All geometry optimizations are done with a 10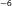 $E_{h}$ threshold for total energy and 3 x 10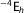/Bohr for energy gradient. Reaction barriers and transition state structures are calculated using the climbing image nudged elastic band procedure^[^ ^37^ ^]^ (NEB‐CI), employing eight image structures between reactant and product structures. Figures from molecular structures are rendered using the VMD 1.9.3 program.^[^ ^38^ ^]^

## Conflict of Interest

The authors declare no conflict of interest.

## Supporting information

Supporting Information

## Data Availability

The data that support the findings of this study are available from the corresponding author upon reasonable request.
