# Supplementary material for: Direct and Indirect Effects of Fe‐Incorporation in Nickel(oxy)hydroxide Materials for the Electrocatalytic Oxygen Evolution Reaction ‐ Employing Constant pH/U Models for Deeper Insights
Source: Chemistry. 2025 Sep 9;31(63):e01441. doi: 10.1002/chem.202501441 (PMC12619057; doi:10.1002/chem.202501441)
Supplement: Supplementary file 1 — Supporting Information [file CHEM-31-e01441-s001.pdf]

Contents

1. PCET diagram for Ni_6FeO_{24} **S2**

2. OER intermediates for all materials: energy, charge and spin multiplicity **S3**

3. OER intermediate free energies **S4**

4. Vibrational spectra - harmonic analysis of OER resting state structures **S5**

5. Size effects in the OER energy profile: comparison between Ni_7O_{24} and $Ni_{19}O_{24}$ LOM mechanism. **S6**
5.1. $Ni_{19}O_{24}$ *OH2*OH intermediate atomic coordinates S6
5.2. $Ni_{19}O_{24}$ *OH*OH intermediate atomic coordinates S8
5.3. $Ni_{19}O_{24}$ *O*OH intermediate atomic coordinates S10

6. Comparison of PBE and B3LYP functionals: $Ni_4Fe_3O_{24}$ OER profile **S13**
6.1. $Ni_4Fe_3O_{24}$ *OH2*OH intermediate atomic coordinates-B3LYP S14
6.2. $Ni_4Fe_3O_{24}$ *OH2*O intermediate atomic coordinates-B3LYP S14
6.3. $Ni_4Fe_3O_{24}$ *OH*O intermediate atomic coordinates - B3LYP S15

7. Atomic coordinates from employed models **S16**
7.1. Molecular structure of the Ni_7O_{24} OER intermediates - pH 14 U=1.1-1.6V S16
7.2. Molecular structure of the Ni_6FeO_{24} OER intermediates - NiNi site - pH 14 U=1.1-1.6V S25
7.3. Molecular structure of the Ni_6FeO_{24} OER intermediates - NiFe site - pH 14 U=1.1-1.6V S33
7.4. Molecular structure of the $Ni_4Fe_3O_{24}$ OER intermediates - NiFe site - pH 14 U=1.1-1.6V S41

8. Metal-oxygen radial distribution function analysis **S50**

1. PCET diagram for Ni_6FeO_{24}

In this section, we report additional data for the proton coupled electron transfer diagram for the Ni_6FeO_{24} material, at pH 14 and U= 1.6 V.

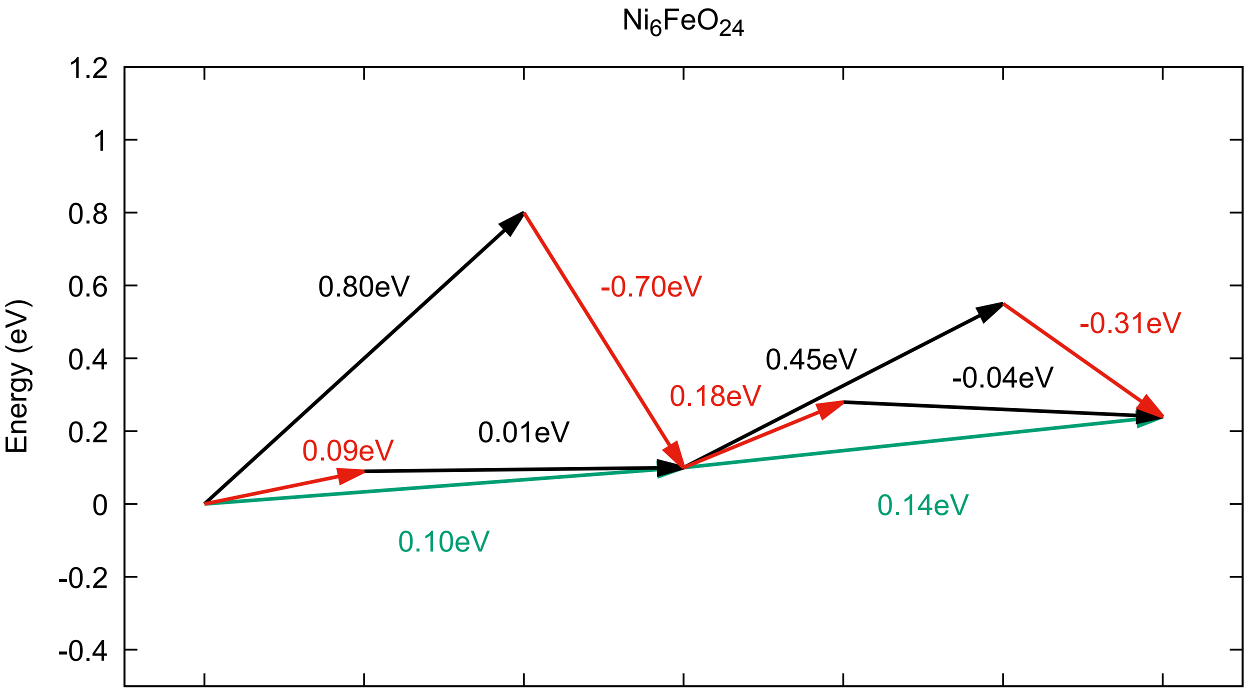

Figure 1. PCET diagram for Ni_6FeO_{24}

2. OER intermediates for all materials: energy, charge and spin multiplicity

Table 1. Raw energies from geometry optimization of all intermediates considered in this study, together with the spin multiplicity and the change in the number of protons and electrons when using the pH/potential protocol.

Ni_7O_{24}	energy (H_a)	delta e	delta H	S^2
*OH2*OH	-12371.0575	(ref)	(ref)	0.76
*OH*OH	-12369.9638	-1	-2	0.00
*O*OH	-12369.9330	-1	-2	2.00
*OO (LOM)	-12369.7610	-2	-2	0.76
*OO (WNA)	-12444.8132	-4	-4	2.05
*OO (IMC)	-12369.1341	-3	-3	2.10
Ni_6FeO_{24} - NiNi site	energy (H_a)	delta e	delta H	S^2
*OH2*OH	-12124.7758	(ref)	(ref)	15.82
*OH*OH	-12124.1009	-1	-1	20.06
*O*OH	-12123.4471	-2	-2	15.83
*OO (LOM)	-12122.8334	-3	-3	20.07
*OO (WNA)	-12198.5360	-4	-4	15.92
*OO (IMC)	-12122.2158	-4	-4	15.93
Ni_6FeO_{24} - NiFe site	energy (H_a)	delta e	delta H	S^2
*OH2*OH	-12125.2122	(ref)	(ref)	15.81
*OH2*O	-12124.7758	0	-1	15.82
*OH*O	-12124.1068	-1	-2	20.06
*OO (LOM)	-12123.9135	-2	-2	15.81
*OO (WNA)	-12200.2671	-2	-2	15.83
*OO (IMC)	-12123.5148	-2	-3	15.96
$Ni_4Fe_3O_{24}$ - NiFe site	energy (H_a)	delta e	delta H	S^2
*OH2*OH	-11635.1399	(ref)	(ref)	20.25
*OH2*O	-11635.1517	0	-0	20.89
*OH*O	-11634.4922	-1	-1	15.86
*OO (LOM)	-11633.8446	-2	-2	20.34
*OO (WNA)	-11709.5249	-3	-3	16.65
*OO (IMC)	-11633.2085	-3	-3	16.58

3. OER intermediate free energies

For the sake of clarity, all free energies from the OER profiles in all materials and mechanisms are reported in this section, as well as the transition state (TS) energy values. These energies come from the data in the SI, section 2 (Unrestricted PBE-D3/def2-SVP/CPCM(water) level of theory), along with the pH/U dependent energy contributions.

Table 2. OER free energies (in eV) for all intermediates considered in this study. TS values are reported in the *OO intermediate in parenthesis, for each mechanism.

Ni_7O_{24}	*OH2*OH	*OH*OH	*O*OH	*OO LOM	*OO WNA	*OO IMC
U = 1.1 V	0.00	0.28	1.12	0.42 (1.55)	-0.96 (1.85)	0.06 (1.95)
U = 1.3 V	0.00	0.08	0.92	0.02 (1.35)	-1.76 (1.65)	-0.54 (1.75)
U = 1.6 V	0.00	-0.21	0.62	-0.57(1.05)	-2.96 (1.35)	-1.44 (1.46)
Ni_6FeO_{24} Ni-Ni	*OH2*OH	*OH*OH	*O*OH	*OO LOM	*OO WNA	*OO IMC
U = 1.1 V	0.00	0.93	1.30	0.57 (1.64)	-1.09 (1.72)	-0.05 (1.75)
U = 1.3 V	0.00	0.73	0.90	-0.02 (1.24)	-1.89 (1.32)	-0.85 (1.55)
U = 1.6 V	0.00	0.43	0.30	-0.92 (0.64)	-3.09 (0.72)	-2.05 (1.25)
Ni_6FeO_{24} Ni-Fe	*OH2*OH	*OH2*O	*OH*O	*OO LOM	*OO WNA	*OO IMC
U = 1.1 V	0.00	-0.17	0.60	0.48 (1.36)	-1.46 (1.57)	-0.71 (1.48)
U = 1.3 V	0.00	-0.17	0.40	0.08 (1.16)	-1.86 (1.37)	-1.11 (1.28)
U = 1.6 V	0.00	-0.17	0.10	-0.51 (0.86)	-2.46 (1.07)	-1.71 (0.98)
$Ni_4Fe_3O_{24}$ Ni-Fe	*OH2*OH	*OH2*O	*OH*O	*OO LOM	*OO WNA	*OO IMC
U = 1.1 V	0.00	-0.32	0.20	0.39 (1.04)	-0.66 (0.50)	0.27 (1.19)
U = 1.3 V	0.00	-0.32	0.00	-0.01 (0.84)	-1.26 (0.30)	-0.32 (0.99)
U = 1.6 V	0.00	-0.32	-0.30	-0.60 (0.54)	-2.16 (0.00)	-1.22 (0.69)

4. Vibrational spectra - harmonic analysis of OER resting state structures

Note that the resting state structures given here have been confirmed as mimima of the geometry optimization using normal mode analysis by computing the Hessian matrix. The corresponding IR spectra are given below:

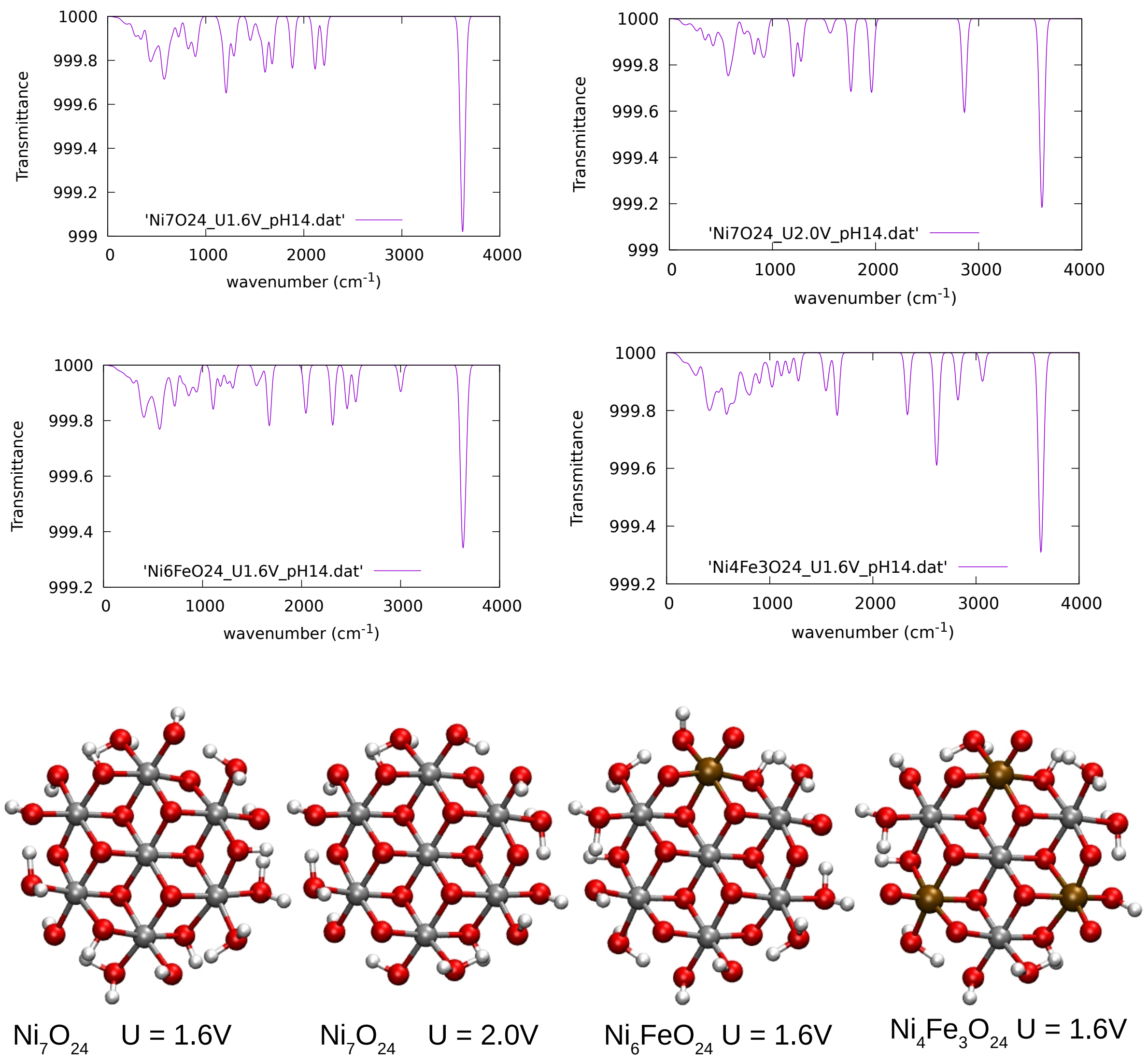

Figure 2. PBE-D3/def2-SCP/CPCM(water) level of theory computed IR spectra for all OER resting state structures studied in this work, together with the corresponding atomic representation of the structural models (lower panel)

5. Size effects in the OER energy profile: comparison between Ni_7O_{24} and $Ni_{19}O_{24}$ LOM mechanism.

In order to assess the influence of the model size in the calculated energies an extra analysis is carried out, where the OER profile for the LOM mechanism is computed using a larger model with an extra layer of metal atoms in the cluster - the $Ni_{19}O_{54}$ model (see Figure 3). Compared to the Ni_7O_{24} model, changes in the charge distribution over are larger when removing electrons and protons, which can be expected from electrostatics. These charge distribution changes will also dictate the resulting acidity of the titratable sites and the redox potential, which can change the stoichiometry of the proton/electron removal. We report the largest difference observed in all calculations, which is found for the second intermediate of the OER reaction in Ni_7O_{24}. Note that the observed changes in the rate-limiting steps are not significant. Hence, we would assume that the Ni_7O_{24} model applied in our study represents a good compromise for optimal computational efficiency and robustness of the most important computational results.

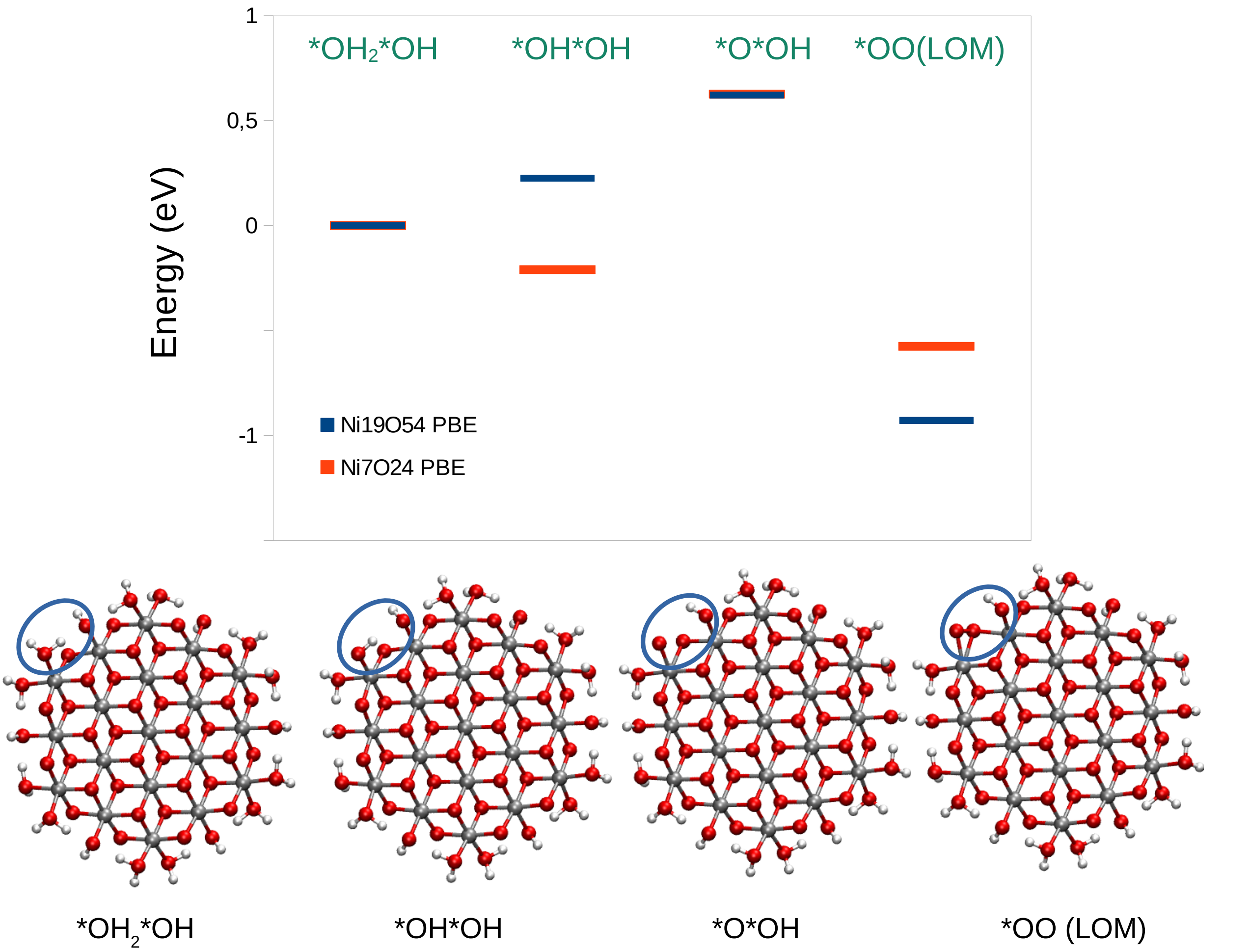

Figure 3. Comparison of OER profile for Ni_7O_{24} and Ni_19O_{24} using the PBE density functional (upper panel). All structures are pH/U optimized and in this case all have zero total charge. Structural models for the intermediates in the large model are shown in the lower panel.

5.1. $Ni_{19}O_{24}$ *OH2*OH intermediate atomic coordinates

Ni 7.33725998486413 10.20628492926195 2.97466400935662
Ni 7.73087997090332 1.76560210781546 2.66749854925030
Ni 9.97262598131901 6.09530471208507 2.98312436203519
Ni 9.79653955471925 8.88621960877690 3.01850894645827
Ni 0.39490614515644 2.85213649984290 2.73173149100129
Ni 2.85462206820115 1.53003617485876 2.79423877395011
Ni 0.21697874061224 5.64161326728144 2.75581148162045

Ni 2.73790905766552 4.35492469531052 2.80826598599489
Ni 2.61080179696761 7.14506380655958 2.90136951377810
Ni 7.58437068655541 4.58447531097260 2.84459940530614
Ni 7.45568101885281 7.37962268655611 2.94470737870018
Ni 5.22724619847821 3.07427218461544 2.78557682192512
Ni 5.09778667498316 5.86621320356809 2.87618576634842
Ni 4.96950026824348 8.65806453723560 2.96676075535908
Ni 2.47564151466435 9.94825999334687 3.03448410553061
Ni 0.14568357562101 8.42170490299904 2.93958052047502
Ni 10.05500803474428 3.30904580078330 2.80304637919834
Ni 4.83172286635433 11.42605101695202 3.08076427426142
Ni 5.35760029584087 0.30931407172553 2.69740261183192
O -0.15257081853955 1.41743479038718 1.51039970281570
O 2.13057890386563 0.12951218870909 1.73605962540764
O 9.24865940099170 2.04677345505384 1.67997599973992
O 8.59671711857612 0.37905459294527 3.62910997872314
O 6.32984152397038 -0.98404215057790 3.79772672306217
O 10.56385487390346 7.49658660645094 4.00887112880201
O 3.29173341499280 11.20180545139298 4.10067706327764
O 11.61726562796524 3.59638505244389 1.65351012807917
O 10.75428637911069 1.85238748109451 3.91508128698812
O 6.88737166107957 0.49689994599110 1.64128519118907
O 11.56874525277265 6.20540997473916 1.96591328457720
O 1.31091412151468 1.59865402404002 3.77782871252127
O 3.80245938600488 0.28038068601534 3.74109412807592
O 4.51571966329678 -1.00423662531493 1.52528550899909
O 5.67122887450004 12.71777588454105 4.27606725292500
O 8.04929739038556 11.59544039524898 4.05585368339811
O 11.37700670661360 8.84303308291569 1.85358625679607
O 10.35447255100012 10.30649092907369 4.25797284664611
O -1.19588788715599 2.91049323468521 3.87546094703884
O 0.94186596556479 9.72596179437389 4.01107186567818
O -0.50756654879860 7.06300384162984 1.83989735356273
O 10.65160485308656 4.67830401762565 3.92586474796899
O -1.42112047379801 8.16567568949048 4.08685556252385
O 6.38604091731369 11.45944055203668 2.03560899222604
O 1.09871620831842 4.24343069659272 3.74654869402416
O 0.97879706465065 7.03627409814055 3.84922828721822
O 6.10730503917141 1.67396392067273 3.70564214148480
O 5.96604693712835 4.47565935819175 3.81200839652680
O 5.83284058812636 7.26726834161257 3.90631627812486
O 5.70718743585274 10.06159681292545 3.98561355159008
O 3.61389223076381 2.97938880851238 3.76783571629172
O 3.47661107098918 5.75510645295340 3.83938691112840
O 3.35355784561232 8.53667129965088 3.95304821007083
O 8.46199117532054 3.17684187328885 3.75390396060579
O 8.30574916558461 5.98581399441854 3.89490053008562
O 8.19758321647561 8.79052676075355 3.96076077577253
O 4.48205374535794 1.66216507042150 1.77733100442750
O 4.36015507318110 4.46438737413415 1.84795488414192
O 4.23152512143810 7.25633164376141 1.93826714299928
O 4.10899171413570 10.06504197156321 2.03464187346861
O 9.21478867774791 4.69682788986882 1.89397017462565
O 9.08888805198829 7.49860968195027 2.00176968815442
O 1.99966528351127 2.93895054623318 1.79497965886852
O 1.88979730440679 5.75105568516065 1.85193410513379
O 1.73256512693696 8.53558555429048 1.97081713350452
O 6.83193078022201 3.19781773192817 1.79725564775926
O 6.71778215796265 5.97893113314220 1.91290665328646

O 6.58481582735920 8.75705944258737 1.98991637199495
O -0.31731595767041 4.24060887515279 1.70284993697075
O 3.83420463072065 12.77370394017442 2.07067214607294
O -1.38553649008029 5.52332267374177 3.76198263429442
O 8.89647787140859 10.18495255418735 2.01308380766066
O -0.62582525834249 9.91372720235628 1.94544298676993
O 1.66659525847427 11.26862845363670 1.92097084806909
H 4.89923547502096 -0.87196861119097 0.62776667590497
H 3.54947640035263 -0.64577752560035 1.46091960118045
H 1.80227044247597 -0.49701189339115 2.42315609998013
H 0.71415887378546 0.85490940218329 1.44299063004450
H -0.78062415606910 0.80922766239943 1.96243156718059
H 7.25438029490548 -0.53382000846223 3.87888367292112
H 6.50173258194973 -1.77360721065288 3.23534087523315
H 8.96332250949781 -0.17777370961364 2.90269096374949
H 9.94294136103797 1.20845311904629 3.94806445943484
H 11.42906221080517 1.34047507275942 3.41373687265502
H -1.98371140341192 2.62249110825786 3.36043274569658
H -2.07243811603295 5.53562930904671 3.05434882686762
H -1.49308384674421 7.13863692675951 4.11522439348141
H -2.20934037573571 8.44162638317703 3.56489138414701
H -0.72655829155778 9.63702327543484 1.00569320897810
H 1.89747187147252 10.93781072291615 1.02028526442076
H 2.89665828642050 12.34044417253240 2.02033725265226
H 3.71275011937475 13.53981338274400 2.67690984086686
H 0.15098078393917 10.59224522381791 1.94505302366559
H 5.29091501144378 12.56895940288780 5.17236401036487
H 8.38908393125212 12.22581815919151 3.37792372100123
H 9.48738050267402 10.86030867903568 4.34228313196197
H 10.97033984896827 10.92555178318048 3.80355484393035
H 6.64076566894935 12.35870743206622 4.33116029957496
H 12.16938030415017 9.12231296919805 2.36666344617029
H 11.51768630098011 7.82619317558482 1.74132588565053
H 12.26128146047035 6.22091498969754 2.66795469401966
H 12.43414693370068 3.34973459757708 2.14422785655462
H 11.66456613909689 4.63109760736158 1.62203588927984
H -1.34050331288853 3.93316015451185 3.97711060790091

5.2. $Ni_{19}O_{24}$ *OH*OH intermediate atomic coordinates

Ni 7.32467532216424 10.20418127017963 2.97712643060975
Ni 7.73490662345450 1.75914573984213 2.65209425926424
Ni 9.96757609004378 6.09374688983289 3.00420282960332
Ni 9.78425247354445 8.88303380859724 3.03366535991866
Ni 0.37297942357175 2.83315500621406 2.73724018105965
Ni 2.85674234454468 1.51542211436679 2.75299882287242
Ni 0.20307440044365 5.63490926441940 2.75863074250003
Ni 2.72569392800093 4.33507312585629 2.80021207745289
Ni 2.60165288151857 7.13066448332050 2.89457156634019
Ni 7.58021699179146 4.57869237292690 2.84931071195775
Ni 7.44619351525377 7.37281358631749 2.95370623963338
Ni 5.22577103161637 3.06481810852451 2.76788440371167
Ni 5.08839752659301 5.85475482652867 2.87619947975225
Ni 4.95810017904770 8.64779701762198 2.96432484828375
Ni 2.46143117700457 9.94058528624105 3.02048157321472
Ni 0.13486711825636 8.40906410067511 2.92176884852258

Ni 10.05315594924470 3.30758038651047 2.81539007770918
Ni 4.81672848650432 11.41909819454724 3.07304843839228
Ni 5.36284270739942 0.29976344493933 2.65707419877597
O -0.20918848820567 1.40789204698748 1.52688017981727
O 2.12887907908504 0.11345865858541 1.69932137771968
O 9.26220300532134 2.04218833254469 1.68172932705239
O 8.59538801681487 0.36533920563802 3.60707880952899
O 6.32984072051495 -1.00259159398651 3.75289370921651
O 10.53031228475277 7.49500216919417 4.04387613367429
O 3.28640734516469 11.17960512580882 4.09731668192055
O 11.61752838246482 3.60060298261705 1.67447356532535
O 10.74500755015137 1.85351307485449 3.93254527277181
O 6.89236234177272 0.51150950770543 1.60266122821680
O 11.57313251179917 6.20970643574902 2.00483074335609
O 1.32979979074468 1.57481581347166 3.73718626307046
O 3.81808545979492 0.27001802016158 3.70296088492980
O 4.51667434847719 -1.00163612955428 1.47699998946481
O 5.65368826535546 12.71463831646818 4.26050902780133
O 8.02897088062855 11.60223915752361 4.04871360460943
O 11.36582325213417 8.83667359675207 1.87894367802348
O 10.33381601170068 10.30078761826200 4.27725402098054
O -1.08301205407788 2.68004043317369 3.82435471813388
O 0.92671099960729 9.71941759682571 3.99366610294829
O -0.48966973424205 7.04855394993830 1.80887137950831
O 10.64037430179309 4.67534609525421 3.94801240832552
O -1.43471223593897 8.14227041971270 4.03787195081179
O 6.35864630918203 11.43140276862674 2.01920543461567
O 1.08910162392944 4.22740248508727 3.74734240183874
O 0.96782136525605 7.02688148627912 3.83761758932102
O 6.10843989804915 1.65739678622284 3.68031638415081
O 5.95709153644470 4.46173475989953 3.80740663379412
O 5.82030333043966 7.26194335582286 3.90772204132876
O 5.69199272065596 10.05680684330379 3.98313961656625
O 3.61206634066599 2.95803277931145 3.74344770980069
O 3.46680202996667 5.74718160502644 3.83615680006957
O 3.33804955797317 8.52801493301767 3.94457881975632
O 8.45278406190235 3.16345645503803 3.75224358800131
O 8.29387674412656 5.97863401442780 3.90653954405844
O 8.17887154567672 8.79280496299326 3.96717726779362
O 4.49312580282168 1.65818999252552 1.74271384988741
O 4.35946456973844 4.45968732510351 1.84237815758648
O 4.22435173725984 7.24556848042279 1.93768124260931
O 4.09709931540886 10.05397462965646 2.02652503379448
O 9.21731669888457 4.69912616507246 1.90889428075223
O 9.08539458774124 7.49200147924793 2.01832717525107
O 2.02660245202293 2.93083996153378 1.76362175373207
O 1.88404019541826 5.73321443670055 1.85137766289086
O 1.73137619147320 8.52839254819207 1.96072196600995
O 6.83786036017240 3.19894700614390 1.78942967537580
O 6.71703204577135 5.97342381969072 1.92019281121200
O 6.57855242443027 8.74945530726583 1.99377014880918
O -0.33091764443833 4.22241945138180 1.73001811766137
O 3.82211281241695 12.76029086359104 2.05654895777822
O -1.38847162224808 5.57809691409711 3.76528348886090
O 8.88383928550672 10.17918687118299 2.01921022089862
O -0.63862805325916 9.89291005798867 1.91997060820382
O 1.65541931483805 11.26082568834116 1.90755920982445
H 4.89643097100978 -0.86855620464990 0.57784619627089
H 3.54689759908796 -0.64508534292223 1.41647485251424

H 1.81961418808830 -0.52241895464855 2.38685321910945
H 0.63111068592275 0.82370397505494 1.45082692894761
H -0.85425642124124 0.86118578618729 2.03094190413157
H 7.25571511974245 -0.55840356554005 3.84288920084057
H 6.49869168248947 -1.79506145931553 3.19343893111032
H 8.98357838883104 -0.18162134234894 2.88414265392355
H 9.93915473781793 1.20448624895592 3.95736845233449
H 11.43168031781714 1.34489886803174 3.44362597920572
H -1.30053069790690 3.62805986858556 4.03787361340940
H -2.08544702751235 5.45773026510422 3.07931870576176
H -1.51346694482645 7.08925185187270 4.02642846573108
H -2.21208051059715 8.44739254781638 3.51582085237565
H -0.73652082773900 9.61345080747140 0.98047713652612
H 1.88375294760030 10.93141087468029 1.00533498217118
H 2.88178478689023 12.33317579191685 1.99961896847557
H 3.70311736394664 13.53736755304776 2.64973779480243
H 0.12873851451545 10.57916702600465 1.91914429068955
H 5.27257650092305 12.57771344839058 5.15867941690388
H 8.38408765059589 12.22996046623259 3.37581837867667
H 9.47304345391597 10.86047536374384 4.35932926154407
H 10.96291049783208 10.91759981472815 3.83767821954740
H 6.62642682708888 12.36142410035888 4.32148058078263
H 12.15744147369347 9.13368712287083 2.38356411237323
H 11.51891828949050 7.81951890627784 1.77331015704718
H 12.26377706881351 6.21868497319952 2.70915661581432
H 12.43527426887564 3.34608194728665 2.16021892396419
H 11.66983266388263 4.63429501274873 1.64657125809793

5.3. $Ni_{19}O_{24}$ *O*OH intermediate atomic coordinates

Ni 7.33661526933934 10.20527217936406 2.94506068580830
Ni 7.73476871753640 1.76299396435004 2.68190979322991
Ni 9.97018973615950 6.09561731117808 3.01164479604938
Ni 9.79594462911346 8.88548792842191 3.01452975293645
Ni 0.40122948339282 2.83020468830281 2.70312340206138
Ni 2.86255215242128 1.52686482985450 2.75394135166028
Ni 0.19441048506369 5.65366004358521 2.86756609544612
Ni 2.72793745063357 4.34584362254941 2.82436933818262
Ni 2.60288411394467 7.13944699109856 2.91519270321444
Ni 7.58220462413713 4.58240261579278 2.86200198463371
Ni 7.45254104817682 7.37698363302862 2.94540165760259
Ni 5.22689591051615 3.07066146777076 2.78572953065175
Ni 5.09094170391163 5.86151782029903 2.88658111929417
Ni 4.96727528904436 8.65332930004104 2.95050368361614
Ni 2.47634724415075 9.95148065850538 3.02285064986856
Ni 0.14198496916861 8.43523689509665 2.99506980524478
Ni 10.05697475615277 3.30760161153697 2.83669239581658
Ni 4.83305673763854 11.42550880692054 3.03665904380630
Ni 5.36455723113691 0.29946061387372 2.66924250624644
O -0.12085759491697 1.44576209860550 1.41568417248310
O 2.16025856821746 0.14372245093197 1.65626231692361
O 9.25488712757105 2.05911703605435 1.70508072939863
O 8.59569188732991 0.38077016719431 3.65089043809275
O 6.31625952094203 -1.00257647362078 3.78216199187206
O 10.55090908486147 7.50608391517674 4.02986975016757
O 3.31345236727579 11.19826147005918 4.07803307393537

O 11.62292213633853 3.59363981780135 1.69737470033341
O 10.74550915917230 1.85524665487517 3.95546513880558
O 6.91709541971685 0.47972367925854 1.65607791642775
O 11.57651569344460 6.19789556172868 2.00999688219326
O 1.29732560683675 1.55160983321619 3.70482611610598
O 3.79711689668774 0.25501777059200 3.69005948526720
O 4.53808432299874 -0.99908332985974 1.47386451790690
O 5.67734663161263 12.73256996107938 4.20513886104468
O 8.04286027259682 11.61162976845312 4.00421416574679
O 11.37696620943291 8.83093845415353 1.85648760776763
O 10.34733364756716 10.31228793131601 4.24642048635158
O -1.20556994576637 2.93586652692913 3.44504359387112
O 0.96407954462349 9.75077112562441 4.03045537412741
O -0.52986447025030 7.06479741701822 1.93292713859406
O 10.62757636083960 4.67835341754688 3.96935639075199
O -1.40652592470513 8.21463885745270 4.13605172971209
O 6.36869496703874 11.42211916951977 1.97536842053487
O 1.09971750926775 4.20606645388788 3.77158550506836
O 1.00151310323107 7.05359507665794 3.89996266313024
O 6.10067532769750 1.65960823450647 3.70055173609912
O 5.96063699732506 4.47171993115206 3.82206309437687
O 5.83038237199989 7.27495901235017 3.90367505172008
O 5.70680541378643 10.06907370309039 3.95538821171439
O 3.61176549452014 2.96264075988160 3.75802062689993
O 3.47270428804123 5.75558933644304 3.85514005225256
O 3.35799755296399 8.54023512139086 3.94440687529262
O 8.45581400733820 3.17536052157253 3.77729281701368
O 8.29649830069348 5.99005078102813 3.91004155295627
O 8.19081614649866 8.80142017760361 3.94787056979698
O 4.50309802621341 1.66486363557760 1.75844299387820
O 4.36028726069986 4.46725683197094 1.85922058683473
O 4.21804128683081 7.24266331623920 1.94125017812388
O 4.10179094207657 10.05211938186508 2.00749320350417
O 9.22011448142321 4.69529563387883 1.92131934346670
O 9.09363556197409 7.48610806905131 2.01146170875939
O 2.04334336570736 2.95451585304264 1.76773489663898
O 1.84899650007974 5.73401507825788 1.89336065829691
O 1.71840504780650 8.53237347049285 1.99034674529146
O 6.83872143749565 3.19778132010993 1.81007976514809
O 6.71705163324464 5.97158024140685 1.92424318182802
O 6.58601666940516 8.74442108568373 1.97431785288057
O -0.52274790947728 4.28618220535337 1.86199704999301
O 3.83158654886096 12.75993168968490 2.01724094724736
O -1.30116808598101 5.68903006899637 3.98935108202332
O 8.90192711648913 10.17743837867188 1.99584061699374
O -0.64392080748356 9.91157372881468 1.99251697464028
O 1.65721492844862 11.26324240737824 1.91076765583642
H 4.93806860879591 -0.86623959987293 0.58330865057912
H 3.57392081840165 -0.63431582995105 1.39443890535687
H 1.81008126523771 -0.49406047271216 2.32225544769120
H 0.73260044816970 0.86656197063917 1.35963210162327
H -0.78521627804755 0.86295267196148 1.84915720392649
H 7.23594188022902 -0.55422638577759 3.88562838696080
H 6.50109285219871 -1.79143670803510 3.22241375442039
H 8.96857345117556 -0.18079948224904 2.93099775054253
H 9.93551233580404 1.20737886491173 3.98168595485593
H 11.43090470345406 1.34376788502604 3.46768811468607
H -2.01448944303049 5.28425810922246 3.44483655296599
H -1.49440724637121 7.14736121210001 4.12560388230414

H -2.18050542353292 8.52509310181145 3.61193364556379
H -0.76275533960142 9.61974001133520 1.05905314482214
H 1.87008227638565 10.92537525673169 1.00791672649955
H 2.89003167481915 12.33493252827649 1.97548088298511
H 3.72110076297708 13.54244287366569 2.60488407214062
H 0.12954067824868 10.59140448219200 1.96610129610891
H 5.29708712003032 12.60872092806047 5.10553226300988
H 8.40085922756911 12.22911421443224 3.32338824637404
H 9.48508812454620 10.87094648248545 4.32343907453517
H 10.97391091836301 10.92638348270181 3.79942204642096
H 6.64950911057192 12.37504151365603 4.26822810233665
H 12.16976098183606 9.12853582548811 2.35897616186896
H 11.52667676802351 7.81422535075645 1.75771478655346
H 12.26888968212696 6.22075094498373 2.71227624900579
H 12.44032832851015 3.33720355956633 2.18269033839737
H 11.67777487112697 4.62845617710494 1.66668841237497

6. Comparison of PBE and B3LYP functionals: $Ni_4Fe_3O_{24}$ OER profile

As semi-local functionals suffer from self-interaction error, additional verification of the functional influence over the intermediate energies is highly advised. Therefore, an additional analysis is performed regarding the employed exchange correlation density functional. For this purpose, the OER intermediates in $Ni_4Fe_3O_{24}$, prior to the O-O coupling reaction, were computed at the B3LYP-D3/def2-SVP/CPCM(water) level of theory (see Figure 4). The pH/potetial protocol is also employed for the determination of total charge and protonation state at this level of theory. The comparison with results from PBE-D3 shows deviations up to 0.3 eV in the calculated energies. As the particular system contains many unpaired electrons (as shown in the SI section 3), introduction of a fraction of exact exchange is expected to yield different energies. The reason it is different for some intermediates is that the number of unpaired electrons for the lowest energy configuration is slightly different for the first intermediate (two extra unpaired electrons compared to PBE), and this affects the energy alignment of the rest of the profile. The B3LYP profile, however, contains very similar qualitative trends regarding the main contributions of Fe incorporation to the NiOx promoted OER. Hence, we would conclude that while the treatment of exact exchange in refined functionals will influence relative energies especially when spin polarization is high, simple functionals like PBE still capture the most important trends.

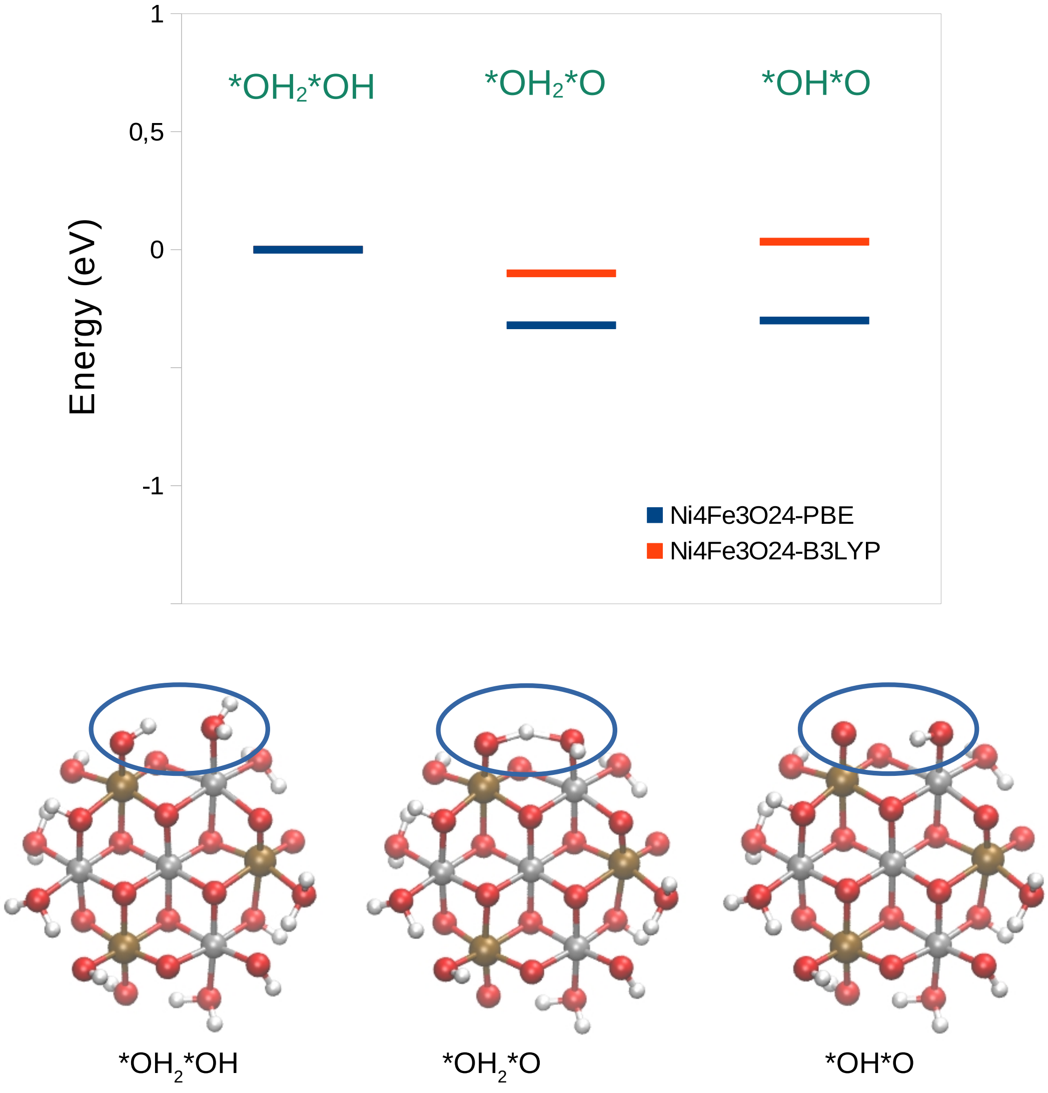

Figure 4. Comparison of the OER profile for $Ni_4Fe_3O_{24}$ using B3LYP and PBE density functionals. All structures are pH/U optimized, and in this case, all have zero charge. Energy reference for SHE and proton solvation energy is -4.44 and -12.2 eV, respectively, together with the pH/U optimized corresponding structures (lower panel).

6.1. $Ni_4Fe_3O_{24}$ *OH2*OH intermediate atomic coordinates-B3LYP

Fe 2.73584457101743 4.26168496245935 12.74719972080456
Ni 2.66629168272508 7.24450175470305 12.94269847861058
Fe 7.60500816582434 4.45211216657972 12.97656173493144
Ni 7.44325603071010 7.36658416322400 12.98141866564988
Ni 5.19001022055517 2.93760961709081 12.87057010560817
Ni 5.09713298893984 5.85715521432953 12.92333323864672
Fe 5.02974239731484 8.72544376627250 12.95892741016118
O 1.04376409721016 4.49871538951331 13.86494242717676
O 1.18341308534107 7.08475479830900 14.05970205230534
O 6.08732406424477 1.48233287439459 14.05752243422396
O 5.97787691175308 4.53811847551629 13.88769794671776
O 5.88300509915586 7.29570797853284 13.92593532083038
O 5.77823202465470 9.94080831893346 14.13409243671799
O 3.38925163520626 3.19522938988886 13.85880176379905
O 3.47573369386011 5.85399504495281 13.83360928283382
O 3.56838326455878 8.55753614265553 13.93794172248564
O 8.30295899889859 3.33030619894615 14.17101591081784
O 8.21702722124734 6.02799314222065 14.08309425795065
O 8.10013971545903 8.83448855533286 14.09125931273202
O 4.29964626529517 1.57183998109532 11.60759787355265
O 4.34280348438536 4.53621442805218 11.87971621249851
O 4.26201231106818 7.27783454320271 11.97779509792758
O 4.25196624329669 9.89487947977524 11.77033113526581
O 9.16420472232979 4.82536332569220 11.98308839799553
O 9.03178613693379 7.34675082538851 11.87845581673481
O 2.14682347912328 3.22512688535904 11.60698268908123
O 1.98867321217586 5.93204866411886 11.79007287232324
O 1.93863315254982 8.72761123881464 11.86651111209834
O 7.03327724859947 3.28533850388771 11.93103200790406
O 6.74196669405009 5.96370145858783 12.02143648365663
O 6.53690195276543 8.62052425138964 11.99081847634295
H 4.67167347008731 1.59121732132680 10.71024276217649
H 4.79192939602105 9.86481312309492 10.95947714065796
H 8.79090569339775 7.68239231042437 10.99587167126229
H 1.22459201415975 4.06729945402488 14.71749309246724
H 5.74757065064090 1.43234631131130 14.96787488767675
H 6.01393174998710 0.57883969662170 13.70445924157181
H 7.24974292041113 9.38500810717060 14.23918933591600
H 5.24865417308352 9.91305776216325 14.95123266243648
H 1.01671832340634 5.97171412995471 11.79593320156160
H 2.77871242416132 9.26882353640863 11.67078379814557
H 3.39319952183597 1.96750688697618 11.51353688689782
H 7.86045188872959 2.46240103644147 14.07909023426959
H 9.19032666936588 6.32100909679306 11.77790005436577
H 9.18750757773333 6.10280688913450 14.06485359976236
H 1.01296778498281 5.50418177751830 14.05415386056133
H 1.39976231912928 9.31644127410088 12.42508884372151
H 0.40872945843846 7.45900998236876 13.60487584469800
H 9.10844944380267 4.32809739881730 11.14982342131881
H 8.67801864873661 9.39516751091431 13.54200208493996

6.2. $Ni_4Fe_3O_{24}$ *OH2*O intermediate atomic coordinates-B3LYP

Fe 2.70366238882825 4.22709515711989 12.84137145005860
Ni 2.65754886957014 7.21250292264873 12.90341410205307
Fe 7.65848483978378 4.40574558058352 12.88934470352512

Ni 7.45917042115234 7.32775350859739 12.99992125874551
Ni 5.19246259361893 3.03513544738296 12.93084165290458
Ni 5.10966308306566 5.84481166530673 12.92510116197503
Fe 5.07859396689802 8.73113780396288 12.93364128369433
O 1.05387798537219 4.49447315043135 13.94165954899335
O 1.16441340779384 7.05906384258202 14.00523699985719
O 6.02990427045206 1.79805366744685 13.99772475101444
O 5.95716693966951 4.47073006177790 13.86806840399850
O 5.88961618793491 7.22521255815396 13.92957156175946
O 5.84908969654592 9.87021418768464 14.19443222191141
O 3.44004823562519 3.20174711128269 13.92713369475299
O 3.48795003693398 5.87762396095322 13.84937515089653
O 3.49199457667268 8.57389554894000 13.91513047490712
O 8.27524184581942 3.23259088043288 14.03716250636930
O 8.21585187108239 5.94631002520815 14.05002048483158
O 8.11461789418159 8.74271024298972 14.15619821820857
O 4.36316416338195 1.67550200436768 11.75386740039654
O 4.35997470179995 4.43051153753546 11.94266812587103
O 4.28203986805347 7.25034261514480 11.97325750470342
O 4.25597614927001 9.86805810217144 12.02019224819966
O 9.18804206192867 4.77143814934853 11.92325741630358
O 9.05216491600548 7.33802015694886 11.90431594442954
O 2.09850111310265 3.16094454182023 11.75486904109112
O 2.04032911375926 5.85022517229698 11.80337642558796
O 1.89790304946526 8.60653723668876 11.73606656946046
O 6.96842777825720 3.33193560662736 11.85072615614446
O 6.74387900909915 5.95592956798505 12.01056998487917
O 6.57255068311317 8.61610639061867 12.01588795746417
H 4.75363858695852 1.72712187131029 10.86256986006083
H 8.83296164958718 7.71133539669998 11.03034886961918
H 1.18834131564343 4.10309676033322 14.82210915184158
H 5.72947976351155 1.99598875902561 14.90270457741119
H 7.25503325828872 9.30421374761593 14.30091884677804
H 5.32300650537803 9.78914569108681 15.01008543845002
H 1.07117606039085 5.90583573215506 11.72076915584798
H 2.67756132596417 9.18421082945219 11.52534942091713
H 3.42830149285210 2.00224482384583 11.64400376143963
H 7.57122292774995 2.51028458857011 14.05901924026608
H 9.22786917942285 6.33764507390302 11.76234820327922
H 9.18314394982714 6.04602118872077 14.10870592149134
H 1.00338801601824 5.51348642234979 14.07818846213462
H 1.27199359376197 9.19615635810849 12.19732835638304
H 0.38154586361335 7.40912513843596 13.54367741142142
H 9.16004030152851 4.28473961605032 11.08137167843848
H 8.71931634325332 9.31176731448239 13.64528239699742

6.3. $Ni_4Fe_3O_{24}$ *OH*O intermediate atomic coordinates - B3LYP

Fe 2.67687986422964 4.23883422360778 12.80255665117735
Ni 2.66938687097174 7.20935099465489 12.91896197646602
Fe 7.66499101867239 4.43242643710556 12.92233795836969
Ni 7.43985495636168 7.31403840423278 13.00870274841110
Ni 5.13612465244089 2.99348575835096 12.98367409594164
Ni 5.09658850982295 5.80877808281835 12.93851405736438
Fe 5.03360822383863 8.68305041031018 12.97229244376481
O 1.01312638104554 4.51453056063111 13.88650565462533
O 1.17448488312912 7.07722284384514 14.01726533156165

O 5.82842678513940 1.73596875891771 14.06769919474784
O 5.96575183402416 4.44029882169051 13.88381653882956
O 5.86981380603006 7.22217459940285 13.93861106128294
O 5.76545752776105 9.87318284065855 14.16235462763012
O 3.37230200770882 3.20446412525162 13.91071587918555
O 3.46944867853732 5.83498485895823 13.84144100013474
O 3.55347653481963 8.52560682887898 13.91733376404130
O 8.34334291309543 3.22034569003843 13.88127276454204
O 8.19445872627507 5.91479052715707 14.08185259721548
O 8.08184751600647 8.73712290668021 14.16595279240084
O 4.29595956875234 1.62553023047607 11.80569849760899
O 4.35884458597917 4.41381021605552 11.94215153103400
O 4.28483479066179 7.21585191527302 11.98213064508882
O 4.28663490306617 9.83989425989514 11.77062273164863
O 9.18513253572763 4.84403631673052 11.90822955811325
O 9.02953835345255 7.35502995028374 11.92459334783646
O 2.07744638506062 3.17678252551600 11.70432796027762
O 2.01965809695439 5.87225164938726 11.78296818770052
O 1.96618265911431 8.66376221321697 11.80432150054387
O 7.05959197303320 3.21645271810602 11.91023526443656
O 6.73209973438158 5.92717785014073 12.02170423327017
O 6.55898285825395 8.57969240997157 12.03291790165513
H 4.70820030097411 1.63410215016132 10.92327823147659
H 4.86346641113784 9.84881887436700 10.98413446609100
H 8.79569586706911 7.73589832563614 11.05772508263961
H 1.13610250098062 4.09889586594367 14.75747268682309
H 6.35186069846391 2.22911001654342 14.72335749772146
H 7.24506992056956 9.30412901473941 14.30821917878259
H 5.20359497916923 9.88184722096781 14.95898571593715
H 1.05083744830924 5.93190517715953 11.70892737633541
H 2.79906838750287 9.21364314567389 11.61908918794604
H 3.37476201619223 1.97509414949636 11.66294709492441
H 9.20377492542379 6.34012857780883 11.77263955849689
H 9.16035850239977 6.00071500762582 14.17709103989720
H 0.98566751756647 5.53135300425176 14.04898013972933
H 1.39226718103612 9.25742961486294 12.32238910608163
H 0.40462938523958 7.44291255195691 13.54644291812204
H 9.11884286065914 4.37562791294621 11.05831620391864
H 8.69828829912517 9.30001724657724 13.66124361722950

7. Atomic coordinates from employed models

In this section, all atomic coordinates of the employed models for evaluating the OER energy profiles are reported. All models are obtained using implicit solvation, with explicit treatment of the total charge and protonation state through the pH/U protocol referred in the main text. Geometry optimizations are performed at the PBE-D3/def2-SVP/CPCM(water) level of theory.

7.1. Molecular structure of the Ni_7O_{24} OER intermediates - pH 14 U=1.1-1.6V

XYZ Coordinates from $Ni_7O_{24}H_{22}^{+}$ - *OH2*OH intermediate

Ni 2.744413 4.380607 12.812897
Ni 2.654638 7.218587 12.848713
Ni 7.588708 4.511654 12.956453
Ni 7.495449 7.387605 13.025063
Ni 5.156730 3.053707 12.814185
Ni 5.120163 5.867510 12.858662

Ni 4.987980 8.662564 12.903022
O 1.177837 4.495202 13.932262
O 1.126781 7.021473 13.917160
O 6.021316 1.783692 13.886438
O 5.938822 4.431894 13.812566
O 5.814937 7.304642 13.890227
O 5.780043 9.966423 14.007417
O 3.646198 3.178122 13.861058
O 3.496892 5.809099 13.782479
O 3.483988 8.453362 13.921476
O 8.209646 3.049688 14.080059
O 8.127604 6.090478 14.224231
O 8.003161 8.859602 14.164689
O 4.248856 1.756881 11.711820
O 4.339211 4.453790 11.835168
O 4.279759 7.260130 11.871521
O 4.024043 9.928431 11.816389
O 9.283212 4.768312 12.051872
O 9.150589 7.321382 12.143087
O 2.076150 2.963671 11.766686
O 1.997158 5.798118 11.700921
O 1.920534 8.628238 11.834251
O 6.693472 3.154863 11.668346
O 6.828922 5.953006 12.017225
O 6.614226 8.592368 11.815245
H 4.622519 1.898051 10.811943
H 4.379942 9.819493 10.904935
H 9.195526 4.457682 11.123470
H 8.967556 7.660102 11.237933
H 1.433837 4.146416 14.816130
H 1.386684 7.394659 14.789278
H 5.550197 1.851319 14.747497
H 7.069229 9.444418 14.172009
H 5.327059 9.859100 14.874004
H 1.013817 5.778704 11.735615
H 3.017389 9.425771 11.786731
H 3.241955 2.210031 11.676195
H 8.912346 2.542012 13.617938
H 9.328416 5.825795 11.991583
H 9.104248 6.210205 14.273068
H 1.088775 5.568390 14.049359
H 1.304773 9.065779 12.464345
H 7.347138 2.423613 14.086259
H 1.517534 2.446108 12.389132
H 7.071480 9.464579 11.838339
H 7.168318 2.295625 11.724721
H 8.689084 9.393591 13.704095

XYZ Coordinates from $Ni_7O_{24}H_{20}$ - *OH*OH intermediate

Ni 2.761892 4.357470 12.820206
Ni 2.657202 7.197570 12.844997
Ni 7.594642 4.565055 12.955505
Ni 7.507576 7.328065 13.000803
Ni 5.202271 3.079995 12.806877
Ni 5.121160 5.867059 12.851680
Ni 5.011536 8.648488 12.887627
O 1.253467 4.307188 13.886680
O 1.135297 7.142912 13.860803

O 6.145118 1.818179 13.937453
O 5.947730 4.478751 13.835273
O 5.838407 7.272581 13.879505
O 5.863641 9.942614 14.066195
O 3.704545 3.168533 13.855600
O 3.476155 5.796041 13.784422
O 3.510322 8.437679 13.908846
O 8.232695 3.200771 14.086750
O 8.090856 5.944781 14.057015
O 8.053173 8.692533 14.169625
O 4.372875 1.772924 11.726860
O 4.388472 4.440682 11.822252
O 4.306199 7.262504 11.864030
O 4.099978 9.920140 11.824892
O 9.263549 4.793237 12.005983
O 9.153098 7.283096 12.073551
O 2.134526 2.873292 11.762319
O 1.942441 5.758559 11.743663
O 1.955018 8.651182 11.800086
O 6.820945 3.243727 11.729391
O 6.788933 5.935724 11.946394
O 6.649816 8.630263 11.818411
H 4.731057 1.965128 10.830485
H 4.482705 9.775252 10.930029
H 9.081040 4.543419 11.072042
H 8.913293 7.572520 11.164387
H 1.601610 3.995347 14.751934
H 5.694427 1.857754 14.810937
H 6.842235 9.499330 14.190314
H 5.421840 9.838858 14.938656
H 0.988562 5.766646 11.997239
H 2.844739 9.302504 11.777192
H 3.063761 2.290974 11.708022
H 8.983571 2.782356 13.609689
H 9.314342 5.900917 11.999592
H 1.079191 6.170123 14.086227
H 1.317302 9.090166 12.406338
H 7.097052 2.335038 14.080932
H 1.555299 2.392029 12.396370
H 7.103233 9.498097 11.910166
H 7.324022 2.400143 11.785341
H 8.793653 9.139744 13.701935

XYZ Coordinates from $Ni_7O_{24}H_{20}$ - *O*OH intermediate

Ni 2.765122 4.294070 12.854760
Ni 2.586117 7.128467 12.904494
Ni 7.589685 4.550659 12.954158
Ni 7.500564 7.314688 12.992100
Ni 5.222605 3.039844 12.816760
Ni 5.101546 5.835942 12.889836
Ni 5.003742 8.642419 12.983338
O 1.248293 4.228543 13.878940
O 1.132276 7.018878 13.908552
O 6.183869 1.772717 13.937025
O 5.961195 4.439630 13.853490
O 5.836601 7.268674 13.901412
O 5.924563 9.964965 14.142238
O 3.725819 3.097268 13.865412

O 3.467966 5.716632 13.833855
O 3.380496 8.352191 14.127414
O 8.255206 3.189247 14.076374
O 8.088835 5.934975 14.047417
O 8.106635 8.666991 14.140369
O 4.402268 1.742067 11.715694
O 4.398819 4.404539 11.857506
O 4.242699 7.232292 11.955807
O 3.986391 9.944305 11.969724
O 9.251734 4.775110 11.983434
O 9.123930 7.271303 12.041993
O 2.159278 2.831705 11.754559
O 1.944034 5.716307 11.787884
O 1.886933 8.543841 11.891512
O 6.836355 3.221722 11.729249
O 6.762267 5.918971 11.955734
O 6.643587 8.616657 11.831154
H 4.766693 1.948785 10.825227
H 4.356066 9.950459 11.059014
H 9.055305 4.522484 11.053198
H 8.858832 7.555224 11.137892
H 1.157903 5.192331 14.148930
H 5.739345 1.806673 14.813530
H 6.889792 9.527586 14.213966
H 5.548106 9.879692 15.045030
H 0.990855 5.636305 12.041457
H 3.036830 9.450729 11.878967
H 3.094148 2.257533 11.694812
H 9.013029 2.788600 13.595416
H 9.299915 5.870003 11.970014
H 1.126785 8.863862 12.429006
H 7.125984 2.300502 14.075483
H 1.566901 2.312955 12.343116
H 7.109385 9.475089 11.947669
H 7.352732 2.387172 11.795593
H 8.855954 9.076321 13.652728
H 2.791848 9.141671 14.152470

XYZ Coordinates from $Ni_7O_{24}H_{20}$ - IMC TS

Ni -2.439654 -1.508578 0.012471
Ni -2.544710 1.289363 0.076150
Ni 2.449696 -1.287359 0.128800
Ni 2.336707 1.481694 0.178755
Ni 0.045860 -2.776077 -0.069989
Ni -0.018590 -0.005723 -0.023067
Ni -0.192286 2.765801 0.056426
O -4.117814 -1.048980 1.315155
O -4.004335 0.669696 1.348800
O 0.961248 -4.058117 1.098077
O 0.793690 -1.389576 0.977965
O 0.665887 1.404772 1.040286
O 0.638274 4.074098 1.242759
O -1.459587 -2.696143 0.983859
O -1.600884 -0.095422 0.931838
O -1.687725 2.539919 1.101975
O 3.071351 -2.660364 1.267275
O 2.928256 0.099362 1.235864
O 2.842094 2.866557 1.360178

O -0.791634 -4.039763 -1.195680
O -0.688433 -1.418984 -1.073237
O -0.854008 1.393426 -0.985941
O -1.139491 4.001968 -1.027391
O 4.140624 -1.038701 -0.806315
O 4.002147 1.459562 -0.730679
O -3.126387 -3.034709 -1.023046
O -3.188396 -0.118599 -1.110155
O -3.343267 2.775491 -0.917108
O 1.717764 -2.622543 -1.109056
O 1.669588 0.087936 -0.899045
O 1.464985 2.782126 -1.006055
H -0.468540 -3.762264 -2.082119
H -0.771508 3.812023 -1.919439
H 3.956671 -1.274511 -1.742681
H 3.761619 1.733683 -1.643509
H -3.755463 -1.164929 2.225750
H 0.488841 -3.981894 1.956446
H 1.628127 3.637407 1.365718
H 0.192804 3.938448 2.108149
H -4.168393 -0.153626 -1.088096
H -2.436810 3.365607 -1.022867
H -2.187658 -3.530227 -1.154243
H 3.826905 -3.073764 0.794837
H 4.174329 0.063973 -0.799759
H -4.917298 0.837341 1.008343
H -3.863636 3.301694 -0.271805
H 1.896074 -3.548434 1.250808
H -3.585484 -3.615808 -0.378675
H 1.903462 3.653391 -0.886319
H 2.220592 -3.459362 -0.996620
H 3.574393 3.329118 0.896211

XYZ Coordinates from $Ni_7O_{24}H_{19}$ - IMC *OO intermediate

Ni 2.739880 4.382233 12.857627
Ni 2.660244 7.215976 12.864460
Ni 7.611968 4.512295 12.986539
Ni 7.534647 7.388770 13.046784
Ni 5.175406 3.072987 12.774941
Ni 5.149928 5.871789 12.861020
Ni 5.017023 8.646975 12.839453
O 1.220976 4.329584 14.071917
O 0.804244 5.456450 14.433033
O 6.028415 1.769920 13.852532
O 5.953013 4.438797 13.825188
O 5.852513 7.316107 13.893985
O 5.799811 10.010424 13.994144
O 3.683711 3.166112 13.843309
O 3.500919 5.820114 13.786324
O 3.519665 8.449345 13.876271
O 8.198634 3.019879 14.102925
O 8.170602 6.097458 14.261244
O 8.034722 8.862663 14.118084
O 4.290568 1.839764 11.630271
O 4.402211 4.489336 11.812151
O 4.395921 7.246211 11.822263
O 4.090772 9.852020 11.711930
O 9.320951 4.765891 12.076289

O 9.204409 7.309881 12.159549
O 2.064875 2.904873 11.800058
O 1.894275 5.800302 11.817189
O 1.853189 8.692907 11.892539
O 6.748652 3.157694 11.655840
O 6.864904 5.941155 12.034532
O 6.696192 8.594280 11.803387
H 4.682399 2.075077 10.759441
H 4.429657 9.610853 10.820561
H 9.208818 4.467403 11.147856
H 9.006801 7.638707 11.255170
H 5.537746 1.849243 14.700136
H 6.797720 9.616984 14.123327
H 5.359689 9.873695 14.862489
H 0.917634 5.778204 11.913264
H 2.762172 9.264071 11.752262
H 3.045594 2.379677 11.643421
H 8.892303 2.510757 13.631472
H 9.360027 5.830316 12.022966
H 9.146751 6.218942 14.269987
H 1.372754 9.230207 12.560260
H 7.309395 2.404690 14.075273
H 1.609637 2.306083 12.431710
H 7.155434 9.458447 11.906531
H 7.203694 2.293691 11.758657
H 8.764190 9.300430 13.625926

XYZ Coordinates from $Ni_7O_{24}H_{20}$ - LOM TS

Ni -2.446604 -1.580243 -0.014255
Ni -2.644519 1.231162 0.061677
Ni 2.360485 -1.343485 0.127172
Ni 2.277356 1.421344 0.183169
Ni -0.004783 -2.852485 -0.027943
Ni -0.121238 -0.052156 0.063673
Ni -0.217034 2.749290 0.115527
O -4.194895 -1.447230 0.800013
O -4.036466 1.222528 1.151020
O 0.955685 -4.131349 1.064126
O 0.731779 -1.461350 1.026640
O 0.597619 1.383925 1.063044
O 0.693989 4.096618 1.283303
O -1.511103 -2.813477 1.014092
O -1.769400 -0.173206 1.003226
O -1.808954 2.503329 1.254999
O 3.021583 -2.725147 1.230402
O 2.846095 0.032508 1.238886
O 2.869983 2.764413 1.343843
O -0.819003 -4.130501 -1.164015
O -0.833235 -1.480998 -0.967612
O -0.986647 1.311552 -0.898161
O -1.224781 4.018150 -0.934693
O 4.027004 -1.112548 -0.835053
O 3.915875 1.381237 -0.737869
O -3.043071 -3.036931 -1.132428
O -3.353849 -0.177319 -0.967211
O -3.325876 2.632390 -0.990362
O 1.604445 -2.651183 -1.117065
O 1.539693 0.029932 -0.865496

O 1.439173 2.720471 -0.997202
H -0.430391 -3.920088 -2.043376
H -0.845113 3.998057 -1.841311
H 3.831901 -1.346098 -1.770523
H 3.670240 1.685441 -1.640873
H -4.088528 -0.694768 1.456119
H 0.506914 -4.112352 1.939072
H 1.646362 3.654477 1.385836
H 0.291354 4.021476 2.175497
H -4.214690 -0.722936 -0.120251
H -2.181509 3.513257 -1.018223
H -2.086335 -3.603186 -1.206011
H 3.774230 -3.123893 0.739663
H 4.083630 -0.017980 -0.829179
H -4.074295 2.985110 -0.457445
H 1.903698 -3.600989 1.212858
H -3.598424 -3.593921 -0.542046
H 1.899961 3.580903 -0.874777
H 2.117586 -3.489091 -1.073445
H 3.640945 3.157036 0.876445
H -2.386840 3.300302 1.256525

XYZ Coordinates from $Ni_7O_{24}H_{20}^+$ - LOM *OO intermediate

Ni 2.733977 4.236534 12.920127
Ni 2.650499 7.156009 12.634430
Ni 7.579425 4.557629 12.923391
Ni 7.491875 7.308195 13.097155
Ni 5.214264 3.048176 12.745391
Ni 5.094205 5.854058 12.873616
Ni 5.031814 8.681501 12.983174
O 1.263650 4.067215 14.185350
O 0.976781 6.354709 12.855597
O 6.203346 1.771876 13.780936
O 5.932570 4.432327 13.806473
O 5.800988 7.232118 13.951036
O 5.911525 9.933963 14.180459
O 3.797291 3.050137 13.883878
O 3.411333 5.764150 13.737359
O 3.443864 8.537763 13.992478
O 8.241203 3.148590 13.984255
O 8.072552 5.887848 14.086941
O 8.038837 8.617294 14.321756
O 4.393620 1.757082 11.644808
O 4.273582 4.420322 11.853385
O 4.356618 7.313020 11.916880
O 4.177146 9.983709 11.927949
O 9.251731 4.817921 11.983206
O 9.138658 7.312742 12.194850
O 2.115905 2.734935 11.863262
O 1.549571 5.516734 11.940386
O 2.050430 8.611770 11.455937
O 6.787288 3.313011 11.635412
O 6.766175 5.963577 11.984013
O 6.638418 8.652745 11.956652
H 4.702289 1.997267 10.741293
H 4.707867 10.000739 11.099876
H 9.074260 4.626770 11.034158
H 8.918633 7.649795 11.296316

H 1.646321 3.977550 15.088860
H 5.755985 1.763904 14.658037
H 6.880117 9.449473 14.328749
H 5.446094 9.857835 15.044331
H 0.847314 4.973916 14.166844
H 2.859162 9.272045 11.542743
H 3.073793 2.217823 11.709571
H 8.987158 2.752671 13.480272
H 9.315934 5.904351 12.040536
H 1.276590 9.082446 11.834538
H 7.178130 2.316074 13.942153
H 1.626154 2.148542 12.482601
H 7.083198 9.525836 12.066559
H 7.297085 2.471861 11.597331
H 8.811623 9.051794 13.894658
H 2.976613 9.397190 13.872915

XYZ Coordinates from $Ni_7O_{25}H_{22}$ - WNA TS

Ni -2.185835 -1.576924 0.007443
Ni -2.329129 1.241071 0.068410
Ni 2.651641 -1.367942 0.142819
Ni 2.604643 1.396542 0.197575
Ni 0.262992 -2.845558 -0.028119
Ni 0.189105 -0.037184 0.065039
Ni 0.105028 2.759693 0.147296
O -3.705628 -1.493248 1.222121
O -3.893783 0.978208 1.021494
O 1.198910 -4.129805 1.084056
O 1.009292 -1.453946 1.023181
O 0.934155 1.382184 1.078797
O 1.035459 4.071221 1.284806
O -1.243042 -2.793913 1.014369
O -1.452955 -0.143965 0.994084
O -1.610647 2.485191 1.315233
O 3.281030 -2.761756 1.255835
O 3.152495 -0.006439 1.261738
O 3.205402 2.746942 1.355703
O -0.553813 -4.115194 -1.172057
O -0.567528 -1.471495 -0.964471
O -0.647012 1.378093 -0.863606
O -0.911245 4.059656 -0.902063
O 4.323775 -1.161730 -0.804632
O 4.255353 1.315602 -0.723219
O -2.811571 -3.059645 -1.100885
O -2.912329 -0.167376 -1.095943
O -3.028097 2.646642 -0.963415
O 1.878405 -2.667907 -1.108020
O 1.860189 0.015722 -0.856429
O 1.847845 2.699047 -1.005661
H -0.172466 -3.883438 -2.049061
H -0.535572 4.045565 -1.809370
H 4.126450 -1.401792 -1.737692
H 4.010146 1.622565 -1.625023
H -3.801113 -0.380847 1.277766
H 0.744315 -4.088938 1.955055
H 1.993867 3.620639 1.377679
H 0.645910 4.005111 2.183532
H -3.938705 -0.075270 -0.906887

H -1.848008 3.563688 -0.991897
H -1.852419 -3.591896 -1.198596
H 4.028179 -3.175959 0.770326
H 4.388125 -0.045526 -0.805356
H -3.771090 3.023729 -0.443044
H 2.158536 -3.609606 1.233684
H -3.324719 -3.650511 -0.506666
H 2.338614 3.539738 -0.875530
H 2.375959 -3.514196 -1.052528
H 3.970444 3.143883 0.883116
H -2.190878 3.276708 1.266474
O -5.225395 0.244862 -0.180496
H -4.527903 -1.725776 0.735368
H -5.535382 1.135483 -0.452332

XYZ Coordinates from $Ni_7O_{25}H_{20}^+$ - WNA *OO intermediate

Ni 2.776116 4.322190 12.808289
Ni 2.616345 7.177028 12.885803
Ni 7.608967 4.527971 12.968433
Ni 7.501041 7.350360 13.024469
Ni 5.225535 3.055054 12.750736
Ni 5.126891 5.843789 12.864300
Ni 4.995809 8.615966 12.865933
O 1.277333 4.119345 13.840542
O 1.032345 7.238177 14.054342
O 6.142508 1.735401 13.845853
O 5.955141 4.437169 13.838490
O 5.836002 7.258278 13.902518
O 5.789184 9.968866 14.020134
O 3.741074 3.107125 13.789928
O 3.471144 5.761619 13.791579
O 3.509565 8.413970 13.888549
O 8.237168 3.148416 14.062371
O 8.174288 5.937132 14.141300
O 8.014571 8.731506 14.164247
O 4.427030 1.792005 11.626743
O 4.411397 4.444539 11.804627
O 4.337472 7.211044 11.855746
O 4.097577 9.835363 11.761805
O 9.285882 4.792897 12.038324
O 9.154845 7.292789 12.122336
O 2.150723 2.869758 11.704744
O 1.865297 5.733183 11.830886
O 1.887880 8.623601 11.827401
O 6.881639 3.289673 11.721355
O 6.807058 5.926335 11.988017
O 6.670899 8.573954 11.835922
H 4.768225 2.027758 10.732806
H 4.475064 9.656340 10.869602
H 9.146051 4.522518 11.102243
H 8.964639 7.608307 11.209921
H 1.605368 4.218249 14.761893
H 5.675501 1.719533 14.712243
H 6.757999 9.570553 14.181931
H 5.328985 9.895855 14.887200
H 0.934185 5.603001 12.131879
H 2.776417 9.224890 11.730953
H 3.056488 2.296754 11.624910

H 9.030953 2.770426 13.619786
H 9.325583 5.889590 12.014003
H 1.301674 9.168722 12.398855
H 7.072714 2.222176 14.034121
H 1.578921 2.377268 12.339567
H 7.139121 9.438477 11.914271
H 7.413897 2.459635 11.753762
H 8.791645 9.156759 13.734585
O 1.158300 6.784150 15.194691
H 7.576922 5.899659 14.928913

7.2. Molecular structure of the Ni_6FeO_{24} OER intermediates - NiNi site - pH 14 U=1.1-1.6V

XYZ Coordinates from $Ni_6FeO_{24}H_{19}$ - *OH2*OH intermediate

Fe 2.601688 4.360929 12.863330
Ni 2.657565 7.147185 12.922487
Ni 7.630193 4.540091 13.002347
Ni 7.495024 7.333040 12.997292
Ni 5.234738 3.077837 13.068647
Ni 5.094996 5.848876 13.021983
Ni 5.093048 8.710506 13.036289
O 1.139629 4.470873 14.048972
O 0.995804 6.998003 13.947364
O 6.129366 1.854028 14.185266
O 5.889395 4.492843 14.056655
O 5.876688 7.275292 14.009747
O 5.995191 10.017838 14.416835
O 3.604422 3.115568 14.101033
O 3.443629 5.818950 13.951207
O 3.456090 8.500356 14.075880
O 8.379199 3.090990 14.185421
O 8.160187 5.924153 14.002382
O 8.206701 8.709236 14.078508
O 4.418139 1.709673 11.878127
O 4.360178 4.407469 12.044484
O 4.249599 7.156679 11.995602
O 4.140306 9.958587 11.965686
O 9.223055 4.767474 11.731515
O 8.998621 7.339626 11.874001
O 2.083769 3.097228 11.919107
O 2.063297 5.726554 11.888228
O 2.061607 8.562663 11.719188
O 6.739453 3.298410 12.037675
O 6.669544 5.938116 12.014279
O 6.584736 8.663886 12.057112
H 4.872551 1.809898 11.010889
H 4.622749 9.991126 11.111402
H 8.894721 4.513778 10.842398
H 8.628743 7.662164 11.021667
H 0.371076 4.043814 13.612157
H 5.733142 2.046780 15.064775
H 6.943279 9.598339 14.341608
H 5.676904 9.697121 15.287633
H 2.931244 9.233114 11.741520
H 3.494225 2.097431 11.721322

H 8.977007 2.551500 13.624259
H 9.228489 5.812013 11.709759
H 0.937345 5.934334 14.051351
H 1.322624 9.058560 12.136186
H 7.507155 2.504595 14.257314
H 8.813558 9.162611 13.450879
H 2.948295 9.339584 13.996549
H 1.195048 7.320255 14.854707
H 3.178295 2.229893 14.070100

XYZ Coordinates from $Ni_6FeO_{24}H_{18}$ - *OH*OH intermediate

Fe 2.606606 4.359159 12.841421
Ni 2.669127 7.143434 12.889727
Ni 7.649653 4.562754 12.968544
Ni 7.511577 7.327647 13.040197
Ni 5.217404 3.055556 13.153016
Ni 5.102501 5.832320 13.036884
Ni 5.105056 8.695352 13.059971
O 1.120235 4.483434 13.988107
O 0.986761 7.005105 13.868812
O 6.035525 1.883898 14.355743
O 5.886035 4.489101 14.087411
O 5.880764 7.249288 14.037712
O 5.961477 9.990553 14.487069
O 3.574751 3.157449 14.138667
O 3.434099 5.819751 13.933816
O 3.449469 8.488688 14.062981
O 8.360364 2.989242 13.706319
O 8.277400 5.910146 13.964600
O 8.200961 8.687322 14.147076
O 4.399653 1.642815 12.019268
O 4.391207 4.376288 12.061194
O 4.280205 7.148870 11.997585
O 4.181422 9.953089 11.986815
O 9.162960 4.819462 11.530518
O 9.008509 7.399822 11.910708
O 2.106514 3.085185 11.904903
O 2.101556 5.719479 11.842525
O 2.095167 8.555792 11.674413
O 6.754294 3.135746 12.147298
O 6.694879 5.926706 12.043984
O 6.622697 8.691512 12.135276
H 4.885493 1.658355 11.163432
H 4.687547 10.004228 11.146777
H 8.734622 4.639307 10.666477
H 8.647023 7.830788 11.103125
H 0.360426 4.047350 13.544458
H 5.742675 2.241253 15.224700
H 6.911492 9.592309 14.425465
H 5.630857 9.653659 15.347519
H 2.952556 9.221923 11.716111
H 3.493318 2.040780 11.802650
H 9.195606 5.850418 11.575351
H 0.918320 5.941923 13.980113
H 1.344411 9.050418 12.072278
H 7.542602 2.533866 14.129275
H 8.836233 9.137890 13.544793
H 2.951524 9.334262 13.981540

H 1.155583 7.337464 14.778992
H 3.156181 2.267215 14.153246

XYZ Coordinates from $Ni_6FeO_{24}H_{17}$ - *O*OH intermediate

Fe 2.575721 4.401576 12.846063
Ni 2.645637 7.192883 12.959860
Ni 7.656865 4.515233 13.128614
Ni 7.462384 7.301680 13.052034
Ni 5.192654 3.094931 13.086888
Ni 5.077360 5.853196 13.069942
Ni 5.055221 8.659011 13.072846
O 1.097631 4.500871 13.999797
O 0.972100 7.020636 13.941366
O 6.272354 1.908378 13.924135
O 5.811128 4.491485 14.104530
O 5.856127 7.269453 14.077135
O 5.921654 9.940485 14.146540
O 3.543893 3.129552 14.089177
O 3.417022 5.846509 13.968914
O 3.437265 8.521811 14.150256
O 8.323666 3.249703 14.306237
O 8.205623 5.940554 14.093727
O 8.138431 8.774366 14.112356
O 4.373606 1.737841 11.901757
O 4.349134 4.446341 12.062851
O 4.281505 7.253098 12.062924
O 4.162370 9.909951 11.977175
O 9.217514 4.747974 11.762552
O 8.964518 7.332603 11.935696
O 2.066143 3.165564 11.866726
O 2.083526 5.791786 11.881218
O 2.027519 8.594910 11.764769
O 6.812946 3.158301 12.190066
O 6.702463 5.873276 12.119851
O 6.532896 8.515753 12.014900
H 4.860172 1.761813 11.046492
H 4.654393 9.858969 11.127223
H 8.839114 4.492043 10.894021
H 8.593510 7.660010 11.084925
H 0.332379 4.073253 13.557133
H 7.233534 9.379202 14.164193
H 5.564457 9.743151 15.042602
H 2.902056 9.241478 11.766044
H 3.466348 2.140709 11.697904
H 9.214872 5.784187 11.739586
H 0.900993 5.953217 14.027270
H 1.304859 9.103667 12.195625
H 8.178286 2.397191 13.827388
H 8.734623 9.241626 13.483921
H 2.956513 9.377709 14.073665
H 1.151598 7.329904 14.858176
H 3.117572 2.243750 14.057016

XYZ Coordinates from $Ni_6FeO_{24}H_{18}$ - IMC TS

Fe -2.434733 -1.602125 -0.191902
Ni -2.398046 1.186454 -0.097845
Ni 2.520073 -1.483038 0.024392

Ni 2.448994 1.324657 -0.008680
Ni 0.191412 -2.929759 0.024316
Ni 0.034092 -0.109228 -0.036635
Ni 0.058350 2.739317 0.014991
O -3.898031 -1.506854 1.018020
O -4.043412 1.006509 0.946117
O 1.425003 -3.989302 1.267242
O 0.847635 -1.439373 0.994764
O 0.821908 1.304804 0.977373
O 0.976791 4.019481 1.417299
O -1.418939 -2.859645 1.056134
O -1.604631 -0.150399 0.908808
O -1.586126 2.524370 1.065064
O 2.928464 -3.143318 1.341331
O 3.054386 -0.093260 1.027931
O 3.175753 2.688617 1.087236
O -0.640681 -4.348727 -1.101127
O -0.713219 -1.528490 -1.021305
O -0.816886 1.215795 -1.041978
O -0.895911 4.003005 -1.037932
O 4.128929 -1.301445 -1.124681
O 3.977834 1.257546 -1.104831
O -2.950809 -2.877739 -1.123943
O -3.004598 -0.230104 -1.141678
O -2.995051 2.630752 -1.270233
O 1.670785 -2.705234 -1.004001
O 1.609530 -0.013542 -1.036351
O 1.564480 2.680032 -0.958819
H -0.187274 -4.328165 -1.973118
H -0.428374 4.029136 -1.900353
H 3.847760 -1.591450 -2.020033
H 3.630652 1.544899 -1.978767
H -4.669755 -1.914545 0.569267
H 1.142161 -3.722842 2.176975
H 1.917577 3.579207 1.344623
H 0.643329 3.689254 2.278641
H -2.113532 3.288945 -1.248950
H -1.550656 -3.943343 -1.279815
H 4.155360 -0.242516 -1.187202
H -4.088102 -0.072579 1.032510
H -3.721251 3.129018 -0.834865
H 3.410009 -3.818059 0.804443
H 3.771595 3.155682 0.459987
H -2.091807 3.364843 0.992501
H -3.824473 1.311937 1.854433
H -1.876567 -3.729179 1.040645

XYZ Coordinates from $Ni_6FeO_{24}H_{15}$ - IMC *OO intermediate

Fe 2.666547 4.411625 12.808242
Ni 2.669413 7.199575 13.032588
Ni 7.669069 4.660784 12.958901
Ni 7.461121 7.433246 12.904220
Ni 5.324083 3.120775 12.905117
Ni 5.132471 5.922207 12.981362
Ni 5.036694 8.735744 13.044548
O 1.241546 4.441405 14.027162
O 1.058571 6.947950 14.086859
O 6.073109 1.662281 14.185708

O 6.036918 4.555992 13.943891
O 5.906616 7.354975 13.986416
O 5.921630 10.017357 14.101268
O 3.696493 3.092016 13.921968
O 3.528284 5.839133 13.959702
O 3.488556 8.530719 14.207835
O 6.558151 2.041958 15.245974
O 8.242045 6.051589 13.905374
O 8.160570 8.890706 13.967862
O 4.676748 1.747256 11.890439
O 4.421253 4.520091 11.960052
O 4.246868 7.320346 12.054473
O 4.056682 9.976666 12.017066
O 9.252322 4.847621 11.829209
O 8.951729 7.401221 11.757771
O 2.085866 3.246198 11.801322
O 2.108859 5.833641 11.920921
O 1.945658 8.613062 11.912470
O 6.990318 3.220988 12.149558
O 6.657348 6.081020 11.920412
O 6.477875 8.656043 11.922277
H 4.497304 9.949450 11.138252
H 9.042845 4.462045 10.949427
H 8.574311 7.644609 10.882190
H 0.468835 4.001983 13.609903
H 7.248537 9.474934 14.069598
H 5.592512 9.809540 15.005873
H 2.799117 9.283222 11.872477
H 3.849410 2.111856 11.489780
H 9.233938 5.898761 11.697334
H 1.013221 5.872748 14.122915
H 1.236096 9.090625 12.397345
H 8.722301 9.395675 13.337169
H 2.978161 9.372634 14.188076
H 1.280623 7.213796 15.008063
H 3.318240 2.201201 13.731502

XYZ Coordinates from $Ni_6FeO_{24}H_{17}$ - LOM TS

Fe -2.442468 -1.686155 -0.113375
Ni -2.353974 1.103622 0.021989
Ni 2.727043 -1.567049 0.233261
Ni 2.463562 1.212899 0.073202
Ni 0.191465 -3.004688 0.048474
Ni 0.080814 -0.251617 0.113080
Ni 0.055412 2.559462 0.101531
O -3.936131 -1.566641 1.041606
O -4.013345 0.933245 1.014196
O 1.459660 -4.180208 0.654314
O 0.818102 -1.640463 1.117822
O 0.867579 1.175973 1.110817
O 0.927816 3.856727 1.154701
O -1.521736 -2.977725 1.086976
O -1.578893 -0.249566 1.016734
O -1.542415 2.433542 1.204665
O 3.413654 -2.698374 1.472118
O 3.228288 -0.089188 1.151132
O 3.138364 2.713818 1.092448
O -0.671478 -4.325876 -1.150360

O -0.675055 -1.630047 -0.903786
O -0.726418 1.152722 -0.888986
O -0.842095 3.807614 -0.993193
O 4.211336 -1.305426 -1.178222
O 3.958578 1.245772 -1.058705
O -2.982913 -2.888062 -1.120010
O -2.933325 -0.275606 -1.063314
O -2.988930 2.509640 -1.161067
O 1.964766 -3.060567 -0.671571
O 1.720147 -0.262615 -0.818166
O 1.515396 2.392191 -0.979566
H -0.219979 -4.304564 -2.023965
H -0.367712 3.737967 -1.851874
H 3.825637 -1.580823 -2.038123
H 3.576875 1.551951 -1.912739
H -4.708947 -1.950151 0.572961
H 2.219718 3.314920 1.155391
H 0.585513 3.665936 2.057827
H -2.113645 3.151513 -1.176772
H -1.581351 -3.917617 -1.321008
H 4.203137 -0.258828 -1.218481
H -4.092986 -0.153299 1.081253
H -3.701527 3.018960 -0.714685
H 2.939308 -3.555807 1.240334
H 3.716314 3.177242 0.444326
H -2.021372 3.290892 1.134535
H -3.817749 1.222234 1.934270
H -1.974040 -3.847881 1.028005

XYZ Coordinates from $Ni_6FeO_{24}H_{16}$ - LOM *OO intermediate

Fe 2.560367 4.460892 12.766713
Ni 2.658409 7.217886 12.941799
Ni 7.798423 4.593119 13.176132
Ni 7.473558 7.344672 13.082046
Ni 4.964657 2.891147 13.005998
Ni 5.099800 5.785348 12.990535
Ni 5.064049 8.629346 13.055634
O 0.997192 4.620456 13.983931
O 1.049423 7.133789 13.921154
O 6.478984 2.356340 11.966077
O 5.906780 4.259621 13.811019
O 5.848906 7.206210 14.044570
O 5.890228 9.920187 14.171235
O 3.411141 3.232261 14.082804
O 3.436717 5.840563 13.906292
O 3.465855 8.509347 14.142867
O 8.703495 3.520555 14.340781
O 8.208215 6.056855 14.180015
O 8.074847 8.808729 14.170078
O 4.124297 1.506757 12.220008
O 4.264334 4.464393 11.940131
O 4.311341 7.239981 12.030751
O 4.193603 9.905138 11.972046
O 9.393567 4.971011 11.903687
O 9.022541 7.488048 12.031859
O 1.913048 3.243597 11.855281
O 2.030724 5.860437 11.851660
O 2.031911 8.634790 11.774843

O 7.570172 3.027104 11.983831
O 6.800718 5.858335 12.116788
O 6.552337 8.523813 12.016037
H 4.670634 9.837496 11.114528
H 9.088673 4.706080 11.008179
H 8.673385 7.796836 11.165075
H 0.200650 4.306991 13.502422
H 7.114761 9.418158 14.210102
H 5.513642 9.722628 15.058926
H 2.903743 9.275176 11.772793
H 3.197069 1.806612 12.020148
H 9.345756 6.018556 11.879249
H 0.903497 5.690297 14.027170
H 1.314512 9.121459 12.239274
H 8.607583 2.598811 14.008403
H 8.682685 9.285525 13.559439
H 2.990458 9.369449 14.078923
H 1.315619 7.419128 14.824553
H 2.894050 2.395890 14.126463

XYZ Coordinates from $Ni_6FeO_{25}H_{19}$ - WNA TS

Fe -2.703502 -1.329587 -0.177146
Ni -2.553031 1.455551 -0.067803
Ni 2.396982 -1.366787 0.217244
Ni 2.256677 1.439420 0.073127
Ni -0.140451 -2.737107 0.132990
Ni -0.157847 0.040374 0.109529
Ni -0.108659 2.855062 0.055130
O -4.207493 -1.178779 0.942124
O -4.247975 1.342379 0.882200
O 0.629296 -3.983947 1.137251
O 0.516342 -1.347355 1.170581
O 0.646477 1.465452 1.094621
O 0.775940 4.135925 1.111627
O -1.827491 -2.634615 1.079039
O -1.841984 0.092451 0.966502
O -1.738174 2.774260 1.119208
O 3.248619 -2.511777 1.374825
O 2.940119 0.103067 1.147777
O 2.975311 2.921288 1.105678
O -0.955220 -4.018603 -1.143284
O -0.904425 -1.342130 -0.904496
O -0.900976 1.446417 -0.933053
O -0.952934 4.110650 -1.075381
O 4.049135 -1.194930 -1.051552
O 3.763638 1.394972 -1.035634
O -3.228929 -2.543770 -1.176285
O -3.136594 0.072168 -1.152273
O -3.114468 2.859562 -1.286044
O 1.494942 -2.624356 -0.734985
O 1.470624 0.001526 -0.819556
O 1.365115 2.653137 -0.998124
H -0.460233 -3.922599 -1.989312
H -0.450522 4.027478 -1.916786
H 3.767877 -1.516410 -1.934502
H 3.397593 1.663810 -1.908657
H -4.978023 -1.574130 0.479008
H 2.090688 3.544643 1.144967

H 0.413126 3.957446 2.009348
H -2.217278 3.482402 -1.285818
H -1.857761 -3.591269 -1.327662
H 4.037771 -0.162524 -1.134615
H -4.354149 0.276324 0.968274
H -3.825992 3.393952 -0.867960
H 4.152252 -2.588275 0.988605
H 3.579721 3.361212 0.465727
H -2.195494 3.641724 1.030585
H -4.078125 1.648830 1.801782
H -2.285444 -3.504361 1.039992
O 3.076029 -4.966671 0.044243
H 3.717301 -5.309763 0.698459
H 2.661598 -4.211738 0.530486

XYZ Coordinates from $Ni_6FeO_{25}H_{17}$ - WNA *OO coordinate

Fe 2.704390 4.263686 12.802496
Ni 2.695013 7.053403 12.969951
Ni 7.762552 4.546961 13.148900
Ni 7.487870 7.319756 12.995467
Ni 5.373924 3.014073 13.027617
Ni 5.165809 5.783489 13.045332
Ni 5.044419 8.600786 13.025917
O 1.230865 4.312523 13.967917
O 1.039634 6.820247 13.972151
O 6.111315 1.613269 14.388422
O 5.988702 4.464369 14.089303
O 5.900283 7.247523 14.028119
O 5.854879 9.956029 14.038816
O 3.691552 2.958591 13.965651
O 3.516107 5.715842 13.954278
O 3.465308 8.396614 14.160251
O 8.781750 3.312322 14.437073
O 8.310467 5.994643 14.036519
O 8.125456 8.840642 14.026379
O 4.805663 1.612621 11.990803
O 4.489088 4.369768 12.017717
O 4.307362 7.159284 12.044030
O 4.117221 9.814586 11.926276
O 9.336478 4.777082 11.856978
O 8.988609 7.352296 11.868824
O 2.164049 3.075720 11.797624
O 2.172758 5.666463 11.867114
O 1.999451 8.464608 11.815325
O 7.042434 3.122454 12.310014
O 6.757787 5.875796 12.074605
O 6.525294 8.493316 11.953927
H 9.017144 4.446824 10.989480
H 8.604552 7.613699 11.001520
H 0.475602 3.872100 13.521361
H 7.218952 9.417212 14.070036
H 5.500861 9.804000 14.944457
H 2.827386 9.159628 11.820849
H 9.295926 5.814723 11.769050
H 0.999881 5.745914 14.029284
H 1.253861 8.915237 12.270249
H 9.029784 2.493355 13.955371
H 8.698112 9.313693 13.380774

H 2.946895 9.231594 14.109386
H 1.227858 7.107266 14.894054
H 3.332742 2.069707 13.734174
O 5.770954 0.446922 14.230984
H 3.968154 1.935546 11.576511
H 4.093359 10.650000 12.443943
H 9.641600 3.732856 14.656258

7.3. Molecular structure of the Ni_6FeO_{24} OER intermediates - NiFe site - pH 14 U=1.1-1.6V

XYZ Coordinates from $Ni_6FeO_{24}H_{20}^+$ - *OH2*OH intermediate

Fe 2.704131 4.396330 12.882350
Ni 2.635875 7.192433 12.988960
Ni 7.636048 4.555071 13.043759
Ni 7.460941 7.361800 12.953199
Ni 5.233208 3.070409 12.955630
Ni 5.107030 5.897695 12.997544
Ni 5.040879 8.692264 13.050265
O 1.239088 4.419500 14.077381
O 1.023813 6.945644 14.046145
O 6.103738 1.684750 14.338247
O 5.909793 4.554503 14.008903
O 5.875004 7.329460 14.010931
O 5.917453 9.977379 14.107960
O 3.614677 3.131254 13.790270
O 3.475527 5.811910 13.948254
O 3.458404 8.503160 14.172303
O 8.467838 3.190518 14.365555
O 8.189521 5.981268 13.970500
O 8.155862 8.821431 14.025128
O 4.421666 1.723409 11.684820
O 4.375229 4.568971 11.939292
O 4.243511 7.295362 12.042068
O 4.050893 9.960885 11.964510
O 9.236845 4.750439 11.823950
O 8.948636 7.346623 11.822144
O 2.094259 3.164495 11.688924
O 2.063159 5.781074 11.932567
O 1.953956 8.574755 11.901565
O 6.833478 3.209762 12.143468
O 6.674724 5.980371 11.992878
O 6.501520 8.588348 11.960041
H 4.859306 1.828155 10.811039
H 4.464176 9.936126 11.071909
H 8.971148 4.411296 10.941224
H 8.573252 7.609158 10.950631
H 0.474777 4.060102 13.573442
H 5.547779 1.606383 15.145060
H 7.257033 9.416770 14.106815
H 5.575320 9.792540 15.012780
H 3.096643 9.445082 11.856157
H 3.497509 2.106211 11.547070
H 9.153850 2.678266 13.884002
H 9.230214 5.784955 11.723621
H 1.009796 5.874672 14.128526

H 1.221492 8.996594 12.403911
H 7.735689 2.528344 14.511083
H 8.740357 9.314507 13.405370
H 2.944446 9.343076 14.145784
H 1.233721 7.259608 14.955214
H 1.729695 3.646758 10.910681
H 6.160858 0.770653 13.981827

XYZ Coordinates from $Ni_6FeO_{24}H_{19}$ - *OH*OH intermediate

Fe 2.601688 4.360929 12.863330
Ni 2.657565 7.147185 12.922487
Ni 7.630193 4.540091 13.002347
Ni 7.495024 7.333040 12.997292
Ni 5.234738 3.077837 13.068647
Ni 5.094996 5.848876 13.021983
Ni 5.093048 8.710506 13.036289
O 1.139629 4.470873 14.048972
O 0.995804 6.998003 13.947364
O 6.129366 1.854028 14.185266
O 5.889395 4.492843 14.056655
O 5.876688 7.275292 14.009747
O 5.995191 10.017838 14.416835
O 3.604422 3.115568 14.101033
O 3.443629 5.818950 13.951207
O 3.456090 8.500356 14.075880
O 8.379199 3.090990 14.185421
O 8.160187 5.924153 14.002382
O 8.206701 8.709236 14.078508
O 4.418139 1.709673 11.878127
O 4.360178 4.407469 12.044484
O 4.249599 7.156679 11.995602
O 4.140306 9.958587 11.965686
O 9.223055 4.767474 11.731515
O 8.998621 7.339626 11.874001
O 2.083769 3.097228 11.919107
O 2.063297 5.726554 11.888228
O 2.061607 8.562663 11.719188
O 6.739453 3.298410 12.037675
O 6.669544 5.938116 12.014279
O 6.584736 8.663886 12.057112
H 4.872551 1.809898 11.010889
H 4.622749 9.991126 11.111402
H 8.894721 4.513778 10.842398
H 8.628743 7.662164 11.021667
H 0.371076 4.043814 13.612157
H 5.733142 2.046780 15.064775
H 6.943279 9.598339 14.341608
H 5.676904 9.697121 15.287633
H 2.931244 9.233114 11.741520
H 3.494225 2.097431 11.721322
H 8.977007 2.551500 13.624259
H 9.228489 5.812013 11.709759
H 0.937345 5.934334 14.051351
H 1.322624 9.058560 12.136186
H 7.507155 2.504595 14.257314
H 8.813558 9.162611 13.450879
H 2.948295 9.339584 13.996549
H 1.195048 7.320255 14.854707

H 3.178295 2.229893 14.070100

XYZ Coordinates from $Ni_6FeO_{24}H_{18}$ - *OH*O intermediate

Fe 2.577487 4.374431 12.849176
Ni 2.653354 7.162620 12.943263
Ni 7.615170 4.523355 13.011644
Ni 7.480793 7.328458 12.965587
Ni 5.214236 3.049460 13.033478
Ni 5.082662 5.844231 13.001566
Ni 5.090918 8.716958 13.031535
O 1.153511 4.480978 14.058381
O 1.001621 7.015481 13.978093
O 6.112738 1.871689 14.219497
O 5.890462 4.516393 14.034343
O 5.875828 7.274314 13.990843
O 5.982057 10.015231 14.436709
O 3.591259 3.045170 14.023676
O 3.450175 5.821573 13.946815
O 3.470234 8.508274 14.091823
O 8.356029 3.128060 14.293917
O 8.224476 5.930295 13.921576
O 8.208211 8.705098 14.028072
O 4.541098 1.671348 12.016311
O 4.345624 4.398869 12.019924
O 4.229640 7.173913 11.993509
O 4.142644 9.978062 11.990372
O 9.194573 4.724816 11.729047
O 8.967955 7.321197 11.818178
O 1.979937 3.178113 11.888256
O 2.054052 5.762985 11.895741
O 2.038878 8.584960 11.755824
O 6.795222 3.243313 12.092918
O 6.648098 5.939020 11.990357
O 6.590811 8.692984 12.066450
H 4.615744 10.023293 11.130856
H 8.869093 4.436768 10.848383
H 8.589439 7.634451 10.965421
H 0.380197 4.022633 13.662948
H 5.660149 2.075011 15.069646
H 6.929839 9.621721 14.349378
H 5.676339 9.674113 15.304795
H 2.895800 9.247158 11.765697
H 3.766075 2.089371 11.563763
H 8.997129 2.572559 13.799072
H 9.203161 5.764742 11.671500
H 0.935231 5.960389 14.080052
H 1.304989 9.081198 12.182055
H 7.489343 2.534341 14.348423
H 8.828654 9.144374 13.402361
H 2.965476 9.351679 14.034803
H 1.198432 7.337200 14.886471
H 3.214909 2.157320 13.809729

XYZ Coordinates from $Ni_6FeO_{24}H_{18}$ - IMC TS

Fe -2.392565 -1.659581 -0.125533
Ni -2.450678 1.129793 -0.125066
Ni 2.517322 -1.485744 -0.025463

Ni 2.391767 1.338164 -0.061525
Ni 0.108685 -2.998275 -0.030291
Ni 0.021793 -0.122925 -0.001941
Ni -0.022740 2.724728 -0.034127
O -3.891394 -1.582691 1.038184
O -4.093264 0.934950 0.919677
O 0.947219 -4.274049 1.067161
O 0.902970 -1.397747 1.032207
O 0.787002 1.320366 0.964485
O 0.870034 4.075318 1.303857
O -1.426173 -2.874603 1.137146
O -1.628963 -0.176000 0.935750
O -1.645047 2.526096 0.996164
O 3.224945 -2.983743 1.250732
O 3.136013 -0.067985 0.884963
O 3.104081 2.733549 1.002753
O -1.328328 -4.071013 -1.191162
O -0.687005 -1.562039 -0.955365
O -0.858759 1.163503 -1.053821
O -1.040204 3.962253 -1.191580
O 4.080282 -1.300220 -1.301198
O 3.878458 1.309143 -1.214578
O -2.847616 -2.971417 -1.065364
O -2.999404 -0.336228 -1.131671
O -3.108408 2.469029 -1.287393
O 1.663407 -2.789185 -0.930024
O 1.554966 -0.016205 -1.055882
O 1.486426 2.698880 -0.964313
H -0.613580 3.971181 -2.075716
H 3.760034 -1.589457 -2.182971
H 3.492284 1.602571 -2.070726
H -4.652503 -2.018767 0.598673
H 0.454298 -4.189458 1.913608
H 1.812625 3.655699 1.258067
H 0.540002 3.802484 2.187012
H -1.947181 3.407539 -1.320871
H -1.176300 -3.805949 -2.128397
H 3.857064 -3.529709 0.735813
H 4.096244 -0.260371 -1.359056
H -4.117100 -0.137077 1.025745
H -3.868691 2.883004 -0.822348
H 2.365474 -3.564530 1.272277
H 3.715474 3.180685 0.374489
H -2.180140 3.346666 0.897539
H -3.886129 1.261161 1.823644
H -1.906697 -3.731793 1.086434

XYZ Coordinates from $Ni_6FeO_{24}H_{17}$ - IMC *OO coordinate

Fe 2.691248 4.502662 13.284115
Ni 2.615339 7.258999 13.010247
Ni 7.550412 4.494173 13.098883
Ni 7.499268 7.357863 13.042837
Ni 5.093772 3.068462 12.924897
Ni 5.109759 5.881243 13.038429
Ni 4.985060 8.705400 12.899370
O 1.305330 4.379974 14.172817
O 0.996406 7.131597 14.242828
O 6.024267 1.690129 13.934737

O 5.916746 4.485722 14.020950
O 5.874530 7.371780 13.948014
O 5.820362 10.047769 13.942993
O 3.525446 3.034694 13.874055
O 3.584834 5.818353 14.132497
O 3.484606 8.517778 13.953741
O 8.158855 3.020861 14.105997
O 8.183682 5.799252 14.296099
O 8.054196 8.870961 14.133476
O 4.362932 1.738689 11.665985
O 4.296833 4.474112 12.045482
O 4.274780 7.213568 11.989386
O 4.069558 9.888352 11.752328
O 9.185835 4.657767 12.166983
O 9.167054 7.187385 12.061604
O 3.234857 1.969310 11.202775
O 2.034329 5.765704 12.117943
O 1.839212 8.642122 11.729511
O 6.746639 3.255992 11.882674
O 6.769227 5.904985 12.112433
O 6.513828 8.676381 11.757339
H 4.513603 9.691092 10.896360
H 9.033346 4.239318 11.291658
H 9.005719 7.489658 11.141140
H 5.623582 1.674832 14.831233
H 7.150404 9.454303 14.134643
H 5.361947 9.974094 14.809329
H 2.709240 9.229192 11.653903
H 8.833420 2.621103 13.512439
H 9.258267 6.121643 11.995739
H 0.875115 6.136987 14.311840
H 1.225942 9.197074 12.257627
H 6.981930 2.170172 14.063832
H 8.722795 9.404828 13.651887
H 1.281488 7.400755 15.143616
H 6.929172 9.564811 11.829780
H 6.482055 3.813891 11.112239
H 9.164501 5.725786 14.258680

XYZ Coordinates from $Ni_6FeO_{24}H_{18}$ - LOM TS

Fe -2.562960 -1.560844 -0.036291
Ni -2.502436 1.197758 -0.132705
Ni 2.449006 -1.501055 0.011949
Ni 2.341080 1.289982 -0.017139
Ni 0.027304 -2.949452 0.029095
Ni -0.069295 -0.156134 -0.023263
Ni -0.037051 2.706222 -0.062367
O -4.031059 -1.332554 1.115741
O -4.142670 1.177538 0.942466
O 0.885007 -4.147805 1.199451
O 0.681308 -1.511200 1.036922
O 0.709722 1.251719 0.961800
O 0.889597 4.050081 1.191602
O -1.750928 -3.061833 0.856574
O -1.690794 -0.157027 0.940620
O -1.671197 2.571451 0.943501
O 3.147437 -2.946232 1.238909
O 3.001341 -0.113992 1.004232

O 3.046454 2.660818 1.064127
O -0.656890 -4.390546 -1.227624
O -0.861819 -1.611494 -0.975771
O -0.903591 1.150344 -1.068689
O -0.988424 3.976509 -1.282389
O 4.090165 -1.288453 -1.216610
O 3.862431 1.290363 -1.115177
O -2.937834 -3.266077 -0.422185
O -3.170612 -0.315186 -1.076054
O -3.126741 2.506786 -1.323941
O 1.634237 -2.736091 -0.980061
O 1.519034 -0.107436 -1.010638
O 1.442840 2.559882 -1.031078
H -0.545740 3.934183 -2.157328
H 3.778802 -1.556042 -2.107895
H 3.507096 1.605957 -1.976277
H -3.806113 -1.753411 1.975220
H 0.479227 -3.942090 2.070916
H 1.829943 3.585894 1.191405
H 0.537738 3.857712 2.087186
H -1.893603 3.470421 -1.410475
H -0.443065 -4.074880 -2.133661
H 3.753963 -3.504343 0.705753
H 4.098728 -0.246926 -1.254642
H -4.211529 0.138231 1.131103
H -3.901842 2.921458 -0.883266
H 2.260486 -3.519272 1.296868
H 3.691301 3.079269 0.449973
H -2.199541 3.391215 0.802325
H -3.921439 1.574887 1.814215
H -1.637074 -4.198304 -1.136429

XYZ Coordinates from $Ni_6FeO_{24}H_{18}^+$ - LOM *OO intermediate

Fe 2.314367 4.630240 12.895001
Ni 2.589313 7.351402 12.934381
Ni 7.527236 4.509615 13.115720
Ni 7.396315 7.330700 13.092616
Ni 5.155194 3.034876 13.080734
Ni 4.963325 5.863851 13.125558
Ni 5.034862 8.700451 13.083656
O 0.833828 4.748452 13.681801
O 0.869455 7.317065 13.872878
O 6.138832 1.768224 14.089854
O 5.697055 4.456998 14.133567
O 5.778237 7.316495 14.097754
O 5.903764 9.990751 14.137580
O 3.645185 2.577382 14.196048
O 3.321377 5.981364 14.036111
O 3.401527 8.637916 14.131415
O 8.303207 3.092436 14.253121
O 8.152503 5.934947 14.213023
O 8.102084 8.796186 14.126582
O 4.701652 1.564582 11.849995
O 4.153251 4.388527 12.199946
O 4.244079 7.291638 12.091298
O 4.193178 9.991485 11.921049
O 9.199033 4.730708 11.935930
O 8.910914 7.308363 12.010651

O 2.479492 2.762029 13.671828
O 1.996485 5.992714 11.791837
O 2.056263 8.723327 11.751061
O 6.707412 3.338291 12.096908
O 6.599966 5.909260 12.179985
O 6.517052 8.529896 12.042824
H 4.665396 9.930881 11.059452
H 8.998958 4.368842 11.046179
H 8.589575 7.647603 11.144700
H 5.740986 1.791553 14.989861
H 7.216185 9.427423 14.167777
H 5.555915 9.818389 15.043037
H 3.212743 9.522148 11.756259
H 3.765237 1.520731 11.553025
H 8.995776 2.631472 13.729604
H 9.182203 5.759139 11.823257
H 0.653133 6.303626 13.851979
H 1.310048 9.194345 12.186196
H 7.481184 2.430681 14.261815
H 8.723730 9.242914 13.506968
H 2.934620 9.502035 14.047149
H 1.049079 7.504675 14.823232
H 4.863499 0.737051 12.360203
H 9.135320 5.996244 14.185193

XYZ Coordinates from $Ni_6FeO_{25}H_{20}$ - WNA TS

Fe -2.366843 -1.319082 -0.192035
Ni -2.332596 1.412767 -0.033353
Ni 2.641434 -1.209269 0.047492
Ni 2.514236 1.576503 0.144413
Ni 0.259084 -2.693906 -0.065614
Ni 0.110264 0.091404 0.035694
Ni 0.122967 2.957963 0.178962
O -3.834344 -1.257199 1.012958
O -3.995883 1.253862 0.963471
O 1.112417 -3.936071 1.069340
O 0.860349 -1.318149 1.023531
O 0.867649 1.471057 1.107149
O 0.992352 4.191880 1.640710
O -1.436094 -2.743339 0.876435
O -1.536473 -0.026459 0.955854
O -1.526887 2.718107 1.189014
O 3.361658 -2.710164 1.187361
O 3.141321 0.130384 1.119237
O 3.204458 2.900595 1.300191
O -0.260935 -4.060257 -1.363892
O -0.616572 -1.308718 -1.036415
O -0.733120 1.451491 -0.929960
O -0.777089 4.264373 -0.852288
O 4.272206 -0.937463 -1.157988
O 4.048900 1.623827 -0.935100
O -3.073303 -2.510044 -1.265206
O -2.941483 -0.017223 -1.174871
O -2.910013 2.920002 -1.189921
O 1.804815 -2.395395 -1.012383
O 1.708539 0.231068 -0.921185
O 1.630066 2.938041 -0.775788
H -0.279442 4.307328 -1.697421

H 3.970203 -1.162476 -2.064210
H 3.703598 1.976351 -1.785930
H -4.596855 -1.662435 0.545151
H 0.687039 -3.732461 1.933061
H 1.942763 3.772673 1.568738
H 0.650972 3.829654 2.486152
H -2.046924 3.557011 -1.133954
H -1.299022 -4.193448 -1.307473
H 3.973948 -3.220282 0.614568
H 4.280141 0.107972 -1.144949
H -4.049337 0.184412 1.057349
H -3.636917 3.412788 -0.750277
H 2.487027 -3.298060 1.204926
H 3.823081 3.382408 0.706140
H -2.034895 3.558596 1.125571
H -3.816328 1.573279 1.875666
H -1.918018 -3.582518 0.677070
O -2.743243 -4.280530 -0.935773
H -0.101025 -3.638265 -2.236972
H -3.307845 -4.582583 -1.683248

XYZ Coordinates from $Ni_6FeO_{25}H_{20}^+$ - WNA *OO intermediate

Fe 2.628806 4.389222 13.012597
Ni 2.532402 7.145183 13.138680
Ni 7.536580 4.532701 13.069179
Ni 7.338081 7.413808 12.937214
Ni 5.170265 3.031148 13.000379
Ni 5.028740 5.873736 13.046425
Ni 4.906661 8.681218 13.099363
O 1.124555 4.320060 14.149550
O 0.956924 6.855939 14.259613
O 6.069359 1.707719 14.427787
O 5.875910 4.549057 14.042228
O 5.795802 7.334801 14.030336
O 5.772331 10.007982 14.110160
O 3.524751 3.023491 13.790230
O 3.422965 5.751006 14.048466
O 3.370034 8.453302 14.298032
O 8.444369 3.177262 14.386236
O 8.171828 6.050752 14.051347
O 8.031206 8.906772 13.968983
O 4.716963 1.375773 11.900514
O 4.280923 4.503914 12.008805
O 4.106751 7.265599 12.137885
O 3.863569 9.932137 12.064767
O 9.117086 4.773675 11.875776
O 8.815883 7.364230 11.797585
O 1.943517 3.161810 11.587895
O 1.964009 5.743272 12.063462
O 1.797839 8.534707 12.090217
O 6.764871 3.208147 12.169765
O 6.574372 5.995642 12.025543
O 6.340044 8.610087 11.962150
H 4.621831 1.583535 10.945318
H 4.234474 9.919239 11.154013
H 8.917016 4.357300 11.009498
H 8.456889 7.636378 10.923661
H 0.341557 4.101264 13.596931

H 5.518543 1.642902 15.239080
H 7.117573 9.478999 14.075719
H 2.895559 9.393529 12.004038
H 3.840899 1.021242 12.168212
H 9.088971 2.642636 13.874176
H 9.068198 5.802708 11.707487
H 0.939779 5.786514 14.304197
H 1.089526 8.952793 12.628645
H 7.707090 2.535238 14.579927
H 8.569665 9.412617 13.319451
H 2.843592 9.284814 14.305018
H 1.208031 7.136964 15.168343
O 2.479490 2.987356 10.483326
H 6.121003 0.788712 14.083414
H 9.148955 6.126483 13.959283
H 5.808551 10.781210 13.503325

7.4. Molecular structure of the $Ni_4Fe_3O_{24}$ OER intermediates - NiFe site - pH 14 U=1.1-1.6V

XYZ Coordinates from $Ni_4Fe_3O_{24}H_{18}$ - *OH2*OH intermediate

Fe 2.727506 4.234046 12.807894
Ni 2.669160 7.200821 12.916858
Fe 7.639929 4.403972 12.937517
Ni 7.463858 7.334120 13.004842
Ni 5.213040 3.058241 12.905763
Ni 5.110897 5.841358 12.928402
Fe 5.034107 8.709437 12.958440
O 1.110044 4.474488 13.947677
O 1.156135 7.015409 14.026625
O 6.037398 1.792064 13.999401
O 5.934930 4.458740 13.912911
O 5.870083 7.260022 13.947870
O 5.790916 9.901884 14.164275
O 3.564754 3.188184 13.889374
O 3.483258 5.812560 13.845901
O 3.544081 8.524879 13.916940
O 8.251614 3.204668 14.073329
O 8.209556 5.939615 14.095504
O 8.107869 8.794118 14.155987
O 4.362661 1.732706 11.722318
O 4.388702 4.445198 11.891070
O 4.285050 7.242314 11.954853
O 4.235153 9.865027 11.756197
O 9.167518 4.760304 11.913921
O 9.076100 7.289150 11.926911
O 2.148524 3.103824 11.725310
O 2.027315 5.812478 11.767585
O 1.943626 8.664342 11.798955
O 6.860819 3.297845 11.900212
O 6.758726 5.932814 12.017055
O 6.562530 8.609124 12.008907
H 4.748308 1.867975 10.827703
H 4.778895 9.835490 10.936140
H 8.836963 7.642386 11.040578
H 1.314138 4.119889 14.839603

H 5.671835 1.991213 14.890646
H 7.228958 9.343335 14.280485
H 5.249239 9.862085 14.984715
H 1.045069 5.827593 11.735111
H 2.805512 9.221531 11.643296
H 3.409470 2.131636 11.643608
H 7.494214 2.500811 14.096885
H 9.197649 6.235774 11.796929
H 9.189329 6.021902 14.121905
H 1.059218 5.535671 14.064047
H 1.397204 9.250612 12.368729
H 0.368064 7.323030 13.526520
H 9.057758 4.334898 11.035947
H 8.676369 9.370054 13.597036

XYZ Coordinates from $Ni_4Fe_3O_{24}H_{18}$ - *OH2*O intermediate

Fe 2.650651 4.445932 12.713400
Ni 2.621168 7.228814 12.999891
Fe 7.595258 4.419507 12.958507
Ni 7.458067 7.339590 13.046974
Ni 5.172968 3.050036 12.958898
Ni 5.084352 5.883970 13.002958
Fe 5.067002 8.761039 12.882070
O 1.114066 4.510471 14.033714
O 1.076428 7.082221 14.064005
O 6.124342 1.691831 14.165322
O 5.888686 4.497395 13.971226
O 5.873311 7.297566 13.994355
O 5.955161 9.979883 14.168589
O 3.518457 3.113910 13.948809
O 3.437278 5.853023 13.925313
O 3.529271 8.554338 14.121380
O 8.246065 3.181669 14.195292
O 8.172329 5.947527 14.155162
O 8.126056 8.763798 14.112611
O 4.317232 1.725459 11.697090
O 4.307996 4.540869 11.942018
O 4.245800 7.295660 12.023846
O 4.276193 9.897439 11.961319
O 8.968214 4.591730 12.019720
O 9.070921 7.213077 11.934333
O 2.080405 3.257364 11.706651
O 1.957572 5.880942 11.932510
O 1.935015 8.672335 11.834084
O 6.713029 3.231577 11.994012
O 6.706987 5.920199 12.068063
O 6.582029 8.601621 12.000782
H 4.799938 1.778870 10.843483
H 8.841052 7.539164 11.035022
H 1.502496 4.197059 14.880224
H 5.720489 1.804515 15.052329
H 6.955024 9.521437 14.216749
H 5.543370 9.789770 15.040611
H 2.752668 9.280657 11.750294
H 3.426668 2.162233 11.508829
H 9.018569 2.741110 13.779368
H 9.161987 6.188743 11.834215
H 9.154089 6.008048 14.150933

H 1.023934 5.553283 14.151197
H 1.258053 9.222061 12.289182
H 7.034293 2.176137 14.251670
H 0.322472 7.203029 13.443890
H 8.695315 9.280558 13.500040
H 2.989938 9.375972 14.148716
H 3.050250 2.254218 13.859810

XYZ Coordinates from $Ni_4Fe_3O_{24}H_{17}$ - *OH*O intermediate

Fe 2.660432 4.268702 12.754151
Ni 2.623127 7.251861 12.895033
Fe 7.599109 4.468104 12.829009
Ni 7.437700 7.364261 12.989269
Ni 5.157184 3.123782 12.933432
Ni 5.070926 5.882349 12.878470
Fe 4.991313 8.738618 12.984242
O 1.104733 4.493512 13.960608
O 1.113935 7.040147 14.009926
O 6.005520 1.903520 14.095488
O 5.875022 4.528412 13.895168
O 5.791468 7.259913 13.932722
O 5.737360 9.898827 14.221573
O 3.508062 3.254390 13.849190
O 3.420730 5.834458 13.765313
O 3.480155 8.549115 13.925051
O 8.186710 3.120535 14.137172
O 8.225329 5.950148 14.022769
O 8.045444 8.780044 14.183806
O 4.383229 1.702504 11.766087
O 4.342969 4.437497 11.857984
O 4.243010 7.297791 11.929517
O 4.182262 9.924553 11.798113
O 8.986606 4.515252 11.945541
O 9.009177 7.493191 12.038903
O 1.959149 3.347122 11.641723
O 1.993706 5.886035 11.736077
O 1.916017 8.731933 11.815939
O 6.735320 3.239888 11.924910
O 6.740770 5.957789 11.966346
O 6.503722 8.668248 12.035027
H 4.943401 1.684220 10.956680
H 4.717537 9.908031 10.972748
H 9.124948 6.593047 11.649220
H 1.352569 4.147217 14.845558
H 5.587583 2.077711 14.968786
H 7.176491 9.344187 14.305871
H 5.200952 9.825580 15.043358
H 1.009765 5.875410 11.724162
H 2.789807 9.298163 11.683419
H 3.512960 2.061638 11.449975
H 8.864906 2.549248 13.714691
H 9.182692 6.081415 13.819107
H 1.037935 5.554771 14.068246
H 1.362115 9.301801 12.395670
H 7.281791 2.507040 14.194001
H 0.318567 7.339376 13.515815
H 8.651175 9.338999 13.646801

XYZ Coordinates from $Ni_4Fe_3O_{24}H_{17}$ - IMC TS

Fe -2.304096 -1.714476 -0.226015
Ni -2.370324 1.285417 -0.090445
Fe 2.571493 -1.381559 -0.090941
Ni 2.460585 1.376131 0.066554
Ni 0.209752 -2.849446 -0.090717
Ni 0.049699 -0.086273 -0.046096
Fe 0.020400 2.766716 -0.003650
O -3.891739 -1.490370 0.976051
O -3.900446 1.090868 0.995240
O 1.080248 -4.082540 1.033945
O 0.878164 -1.473227 0.946750
O 0.751393 1.321471 0.999298
O 0.781189 3.934255 1.226123
O -1.437329 -2.757657 0.845260
O -1.593737 -0.123516 0.828334
O -1.508891 2.617196 0.912582
O 3.274220 -2.804240 1.126081
O 3.055197 -0.008695 1.254379
O 3.147843 2.857119 1.186927
O -0.597409 -4.225609 -1.297375
O -0.635693 -1.508296 -1.112371
O -0.743912 1.314865 -1.034034
O -0.751184 3.934727 -1.234837
O 4.040967 -1.035286 -0.887478
O 4.134122 0.714271 -1.042071
O -2.963876 -2.707259 -1.332061
O -3.001798 -0.095862 -1.241055
O -3.048906 2.766813 -1.206704
O 1.840851 -2.615365 -1.084050
O 1.672808 -0.000768 -0.956830
O 1.573384 2.647118 -0.929200
H -0.134004 -4.151851 -2.161748
H -0.212650 3.876234 -2.055663
H 3.859459 0.740704 -1.989964
H -3.634681 -1.807732 1.868343
H 0.660432 -3.914888 1.907298
H 2.261800 3.376101 1.329709
H 0.246716 3.851714 2.048007
H -3.984896 -0.102195 -1.227636
H -2.165363 3.311653 -1.341810
H -1.510301 -3.853910 -1.482307
H 3.944330 -3.334046 0.643197
H 4.037495 0.014206 1.296430
H -3.967155 -0.441016 1.065613
H -3.587293 3.354492 -0.630650
H 2.403954 -3.427045 1.156241
H -4.680832 1.386366 0.476148
H 3.670013 3.454689 0.607199

XYZ Coordinates from $Ni_4Fe_3O_{24}H_{15}$ - IMC *OO intermediate

Fe 2.655550 4.262784 12.794933
Ni 2.666535 7.217725 12.876404
Fe 7.466139 4.293418 13.230939
Ni 7.490722 7.284274 13.053773
Ni 5.044816 2.966214 13.035612
Ni 5.089504 5.799335 13.025544

Fe 5.059007 8.658927 12.964813
O 1.022702 4.558102 13.912787
O 1.111039 7.103084 13.934401
O 5.723435 1.511022 14.221440
O 5.869596 4.422022 14.116376
O 5.801790 7.231675 14.012635
O 5.805807 9.871901 14.152695
O 3.401833 3.159602 13.929923
O 3.419983 5.826367 13.863925
O 3.522521 8.548157 13.869993
O 8.605337 3.534400 14.021006
O 8.187713 5.961088 14.198972
O 8.141709 8.798365 14.129387
O 4.160674 1.622715 11.773425
O 4.339329 4.386502 11.992561
O 4.320228 7.165686 11.976424
O 4.297222 9.788958 11.689924
O 9.573899 6.199859 11.658768
O 9.082077 7.317862 11.921321
O 2.029960 3.152795 11.722975
O 2.021426 5.832788 11.725184
O 2.009463 8.661403 11.704128
O 6.705238 3.198261 12.167830
O 6.810762 5.800300 12.162079
O 6.580210 8.527386 12.021754
H 4.565113 1.752136 10.888164
H 4.863765 9.723474 10.888466
H 1.223040 4.218550 14.811569
H 5.870885 1.866349 15.126038
H 7.251348 9.322230 14.267183
H 5.264043 9.831846 14.972839
H 1.040417 5.863114 11.679330
H 2.903046 9.194607 11.562914
H 3.240839 2.059954 11.678597
H 9.170359 5.935733 14.183028
H 0.985598 5.618875 14.007732
H 1.473121 9.271711 12.257990
H 6.615372 1.220274 13.928827
H 0.349918 7.411915 13.394923
H 8.670162 9.392074 13.551126

XYZ Coordinates from $Ni_4Fe_3O_{24}H_{17}$ - LOM TS

Fe -2.271202 -1.696549 -0.286551
Ni -2.322873 1.258194 -0.077937
Fe 2.548008 -1.584513 0.167845
Ni 2.497869 1.356392 0.063423
Ni 0.159245 -2.941921 -0.076668
Ni 0.106128 -0.124012 -0.024840
Fe 0.057079 2.738401 0.031438
O -3.904668 -1.484599 0.853780
O -3.861073 1.065328 0.995342
O 0.961002 -4.285677 1.145380
O 0.856919 -1.483909 1.015556
O 0.899620 1.262263 1.010515
O 0.803355 3.909495 1.264316
O -1.491011 -2.828822 0.809379
O -1.544633 -0.155573 0.841112
O -1.447761 2.540814 0.971161

O 3.043996 -2.826062 1.469636
O 3.423813 0.026871 1.037997
O 3.111891 2.803360 1.246314
O -0.699344 -4.276158 -1.355252
O -0.610223 -1.499414 -1.097764
O -0.684209 1.292736 -1.007405
O -0.729975 3.924151 -1.149350
O 4.131274 -1.068159 -0.230189
O 4.105032 1.459303 -1.066996
O -2.873187 -2.784438 -1.394080
O -2.961777 -0.086600 -1.280213
O -3.019186 2.745934 -1.180925
O 1.849803 -2.764988 -0.912985
O 1.799082 -0.089848 -0.885033
O 1.572757 2.652850 -0.913991
H -0.297691 -4.124732 -2.238440
H -0.168424 3.917673 -1.957941
H 3.809488 1.579543 -1.998481
H -3.692044 -1.863692 1.733904
H 0.449210 -4.220456 1.980679
H 2.227850 3.346807 1.375963
H 0.252400 3.857169 2.077715
H -3.944283 -0.078589 -1.294321
H -2.149732 3.301889 -1.304263
H -1.627658 -3.857508 -1.442836
H 3.858607 -3.272724 1.148263
H 4.398937 0.500061 -1.005348
H -3.957226 -0.433298 1.001340
H -3.576681 3.322020 -0.611892
H 1.863515 -3.807487 1.363555
H -4.635994 1.383941 0.481770
H 3.683976 3.394535 0.707319

XYZ Coordinates from $Ni_4Fe_3O_{24}H_{16}$ - LOM *OO intermediate

Fe 2.784360 4.221789 12.753663
Ni 2.695795 7.179329 12.924725
Fe 7.627744 4.424299 13.141162
Ni 7.510851 7.436693 12.976907
Ni 5.263286 3.061585 12.914031
Ni 5.162043 5.859253 12.934347
Fe 5.030711 8.726132 13.037639
O 1.146322 4.423368 13.859501
O 1.169652 6.949411 14.011423
O 6.061196 1.789822 14.022348
O 5.912910 4.495818 13.977394
O 5.954258 7.255872 13.969714
O 5.760437 9.894274 14.277656
O 3.592886 3.154620 13.839162
O 3.522795 5.789993 13.829236
O 3.544938 8.468159 13.986469
O 8.177573 3.236443 14.301415
O 8.594639 6.066536 13.924366
O 8.112206 8.873930 14.179905
O 4.457340 1.745107 11.682058
O 4.471773 4.449959 11.887150
O 4.327579 7.268204 11.983765
O 4.189002 9.909625 11.891866
O 9.280715 5.327319 12.979181

O 9.051754 7.680154 11.793744
O 2.236301 3.106638 11.640869
O 2.093852 5.812528 11.729072
O 1.953265 8.650851 11.837561
O 7.014240 3.333784 12.004435
O 6.865547 5.953375 12.071504
O 6.514516 8.712566 12.046193
H 4.864298 1.895337 10.799384
H 4.745281 9.942547 11.080141
H 8.709331 7.836989 10.884003
H 1.328927 4.041507 14.744722
H 7.219742 9.384919 14.347821
H 5.231850 9.806613 15.102972
H 1.112318 5.816509 11.679952
H 2.803194 9.241068 11.725121
H 3.504414 2.140195 11.584106
H 7.436560 2.498271 14.253517
H 9.467815 6.775349 11.766785
H 1.085901 5.484150 14.006407
H 1.370173 9.201782 12.406050
H 5.606255 1.911354 14.885664
H 0.380587 7.254568 13.511336
H 8.656653 9.499983 13.652180

XYZ Coordinates from $Ni_4Fe_3O_{25}H_{19}$ - WNA TS

Fe -2.555617 -1.480960 -0.334101
Ni -2.595620 1.312246 -0.183018
Fe 2.322480 -1.434600 0.166899
Ni 2.243065 1.522121 0.077787
Ni -0.039251 -2.831609 0.058306
Ni -0.115818 0.002555 -0.020413
Fe -0.172053 2.873598 -0.240298
O -4.135695 -1.359972 0.910868
O -4.196843 1.185412 0.808870
O 0.869596 -4.107981 1.321172
O 0.663199 -1.276787 1.087043
O 0.616888 1.464800 0.947982
O 0.608665 4.145779 1.051379
O -1.727786 -2.753895 0.981876
O -1.799750 -0.017734 0.830419
O -1.772449 2.680002 0.937100
O 2.982686 -2.671904 1.416442
O 2.978642 0.156162 1.222625
O 2.785388 2.996962 1.176548
O -0.836815 -4.234665 -1.234883
O -0.832322 -1.424994 -1.038950
O -0.928455 1.370761 -1.083568
O -0.970428 3.970057 -1.217726
O 3.919422 -1.501082 -0.709990
O 3.900849 1.613933 -0.972596
O -3.087116 -2.717965 -1.302660
O -3.173670 -0.097670 -1.230440
O -3.249810 2.703421 -1.429491
O 1.531651 -2.633135 -0.841039
O 1.578969 0.021018 -0.884035
O 1.383634 2.739385 -1.030029
H -0.325007 -4.141243 -2.066740
H -3.776178 -1.629219 1.784968

H 0.428452 -3.990690 2.189915
H 1.634830 3.704062 1.169487
H 0.153284 3.963189 1.902876
H -2.426506 3.318271 -1.489503
H -1.721137 -3.802401 -1.445433
H 3.722653 -3.154355 0.987687
H 2.942885 -0.861418 -2.250117
H 3.956339 0.185641 1.120877
H -4.239976 -0.304812 0.971037
H -3.946726 3.260336 -1.014911
H 1.790796 -3.613061 1.439701
H -4.920838 1.284164 0.150744
H 3.410282 3.519545 0.627752
H -2.326345 3.491913 0.908759
H -2.182726 -3.621201 0.900442
O 3.900398 -0.816599 -1.984108
H 3.992750 0.722003 -1.460755
H 3.737135 2.272615 -1.684755

XYZ Coordinates from $Ni_4Fe_3O_{25}H_{17}$ - WNA *OO intermediate

Fe 2.604465 4.230708 12.889909
Ni 2.673475 7.189615 12.853182
Fe 7.473510 4.244011 12.824141
Ni 7.479062 7.173617 12.908272
Ni 5.025371 2.964014 13.043870
Ni 5.070853 5.745417 12.882870
Fe 5.080264 8.624820 12.883889
O 1.026368 4.591869 14.034836
O 1.155854 7.105230 13.975405
O 5.825218 1.765023 14.247109
O 5.838533 4.383867 13.930123
O 5.899676 7.128984 13.860260
O 5.794052 9.831682 14.041705
O 3.405813 3.216854 14.036047
O 3.456943 5.821050 13.829231
O 3.575359 8.496228 13.826042
O 8.046534 2.938080 14.230929
O 8.337454 5.690000 13.826158
O 8.225226 8.427803 14.022570
O 4.104932 1.578211 11.998645
O 4.255214 4.309818 11.940642
O 4.292645 7.138810 11.886633
O 4.303786 9.751139 11.623611
O 9.969857 4.790992 11.735130
O 9.109235 7.392975 11.849336
O 1.969206 3.059165 11.884373
O 1.977031 5.767445 11.770420
O 1.998973 8.627234 11.689804
O 6.620965 3.005952 11.995623
O 6.732792 5.687479 11.898058
O 6.606164 8.478673 11.951743
H 4.496799 1.600219 11.096153
H 4.833417 9.637142 10.801917
H 9.522817 8.049859 12.467699
H 1.235423 4.280247 14.941998
H 5.392645 1.975191 15.105258
H 7.503405 9.106312 14.124621
H 5.194290 9.858745 14.822599

H 0.998359 5.822474 11.697876
H 2.888104 9.157135 11.531966
H 3.172588 2.012847 11.878457
H 8.700183 2.303631 13.863465
H 8.041491 5.706070 14.763789
H 1.013497 5.672715 14.088365
H 1.461892 9.242894 12.236969
H 7.133801 2.365142 14.330071
H 0.374857 7.405500 13.460021
O 9.043616 3.942930 11.843313
H 9.620078 6.541802 11.963619

8. Metal-oxygen radial distribution function analysis

In order to highlight the structural distortion in the optimized models that occur upon Fe incorporation, metal-oxygen radial distribution functions (RDF) are reported for all resting state structures in Figure 5. Note that a broadening of the first RDF peak can be observed due to the shorter Fe-O bond lengths, as well as the formation of oxo groups.

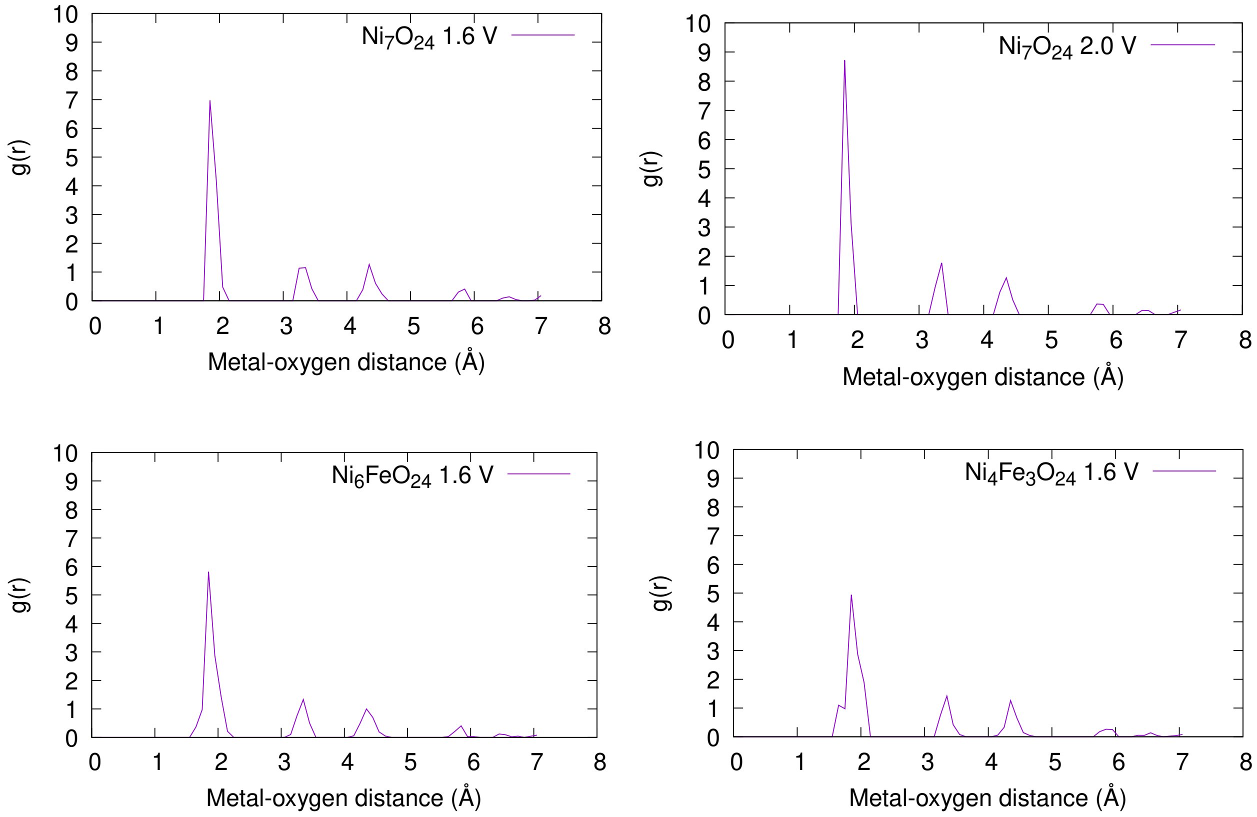

Figure 5. Metal-oxygen radial distribution functions for all resting state structures considered in this study.
